# A comprehensive review on NUI, multi-sensory interfaces and UX design for applications and devices for visually impaired users

**DOI:** 10.3389/fpubh.2024.1357160

**Published:** 2024-10-25

**Authors:** Lauryn Arora, Akansh Choudhary, Margi Bhatt, Jayakumar Kaliappan, Kathiravan Srinivasan

**Affiliations:** ^1^School of Computer Science Engineering and Information Systems, Vellore Institute of Technology, Vellore, India; ^2^School of Computer Science and Engineering, Vellore Institute of Technology, Vellore, India

**Keywords:** user experience, user interface, human–computer interaction, visually impaired, digital health

## Abstract

In today’s world, there has been a significant increase in the use of devices, gadgets, and mobile applications in our daily activities. Although this has had a significant impact on the lives of the general public, people who are Partially Visually Impaired SPVI, which includes a much broader range of vision loss that includes mild to severe impairments, and Completely Visually Impaired (CVI), who have no light perception, still face significant obstacles when trying to access and use these technologies. This review article aims to provide an overview of the NUI, Multi-sensory Interfaces and UX Design (NMUD) of apps and devices specifically tailored CVI and PVI individuals. The article begins by emphasizing the importance of accessible technology for the visually impaired and the need for a human-centered design approach. It presents a taxonomy of essential design components that were considered during the development of applications and gadgets for individuals with visual impairments. Furthermore, the article sheds light on the existing challenges that need to be addressed to improve the design of apps and devices for CVI and PVI individuals. These challenges include usability, affordability, and accessibility issues. Some common problems include battery life, lack of user control, system latency, and limited functionality. Lastly, the article discusses future research directions for the design of accessible apps and devices for visually impaired individuals. It emphasizes the need for more user-centered design approaches, adherence to guidelines such as the Web Content Accessibility Guidelines, the application of e-accessibility principles, the development of more accessible and affordable technologies, and the integration of these technologies into the wider assistive technology ecosystem.

## 1 Introduction

The all-pervasive concept of interface design was introduced in the 2000s and has seen development into the age of the Internet of Everything. Interface technologies, namely User Interface (UI) and User Experience (UX), have evolved into Character User Interface (CUI), Graphical User Interface (GUI), and Natural User Interface (NUI) because of the emergence of cloud computing, internet of things, and artificial intelligence that are employed in mobile applications (1). The formal definition of user experience given by the International Organization for Standardization (ISO) as per ISO 9241-210 is: “User’s perceptions and responses that result from the use and/or anticipated use of a product, system, or service” (2). According to Wikipedia “In computing, a natural user interface (NUI) or natural interface is a user interface that is effectively invisible and remains invisible as the user continuously learns increasingly complex interactions. The word “natural” is used because most computer interfaces use artificial control devices whose operation must be learned. Examples include voice assistants, such as Alexa and Siri, touch and multitouch interactions on today’s mobile phones and tablets, but also touch interfaces invisibly integrated into the textiles furniture” (3).

The World Health Organization (WHO) estimates that 2.2 billion individuals worldwide have some form of vision impairment, either near or farsightedness. A significant number of these instances could have been avoided or are still not addressed because of eyesight impairment. These numbers indicate that there is still a need to create highly interactive, useful, and easy-to-understand interfaces to help the visually impaired community live an easier life (4, 5). Visual impairment can have several root causes, including uncorrected refractive defects, age-related macular degeneration, cataracts, glaucoma, diabetic retinopathy, amblyopia, high myopia and retinoblastoma (6, 123).

This study investigates the essential elements in producing technological products for two categories of individuals. Firstly, Completely Visually Impaired (CVI): These are people with no functional vision, and they cannot perceive any form of light. CVI people cannot see shades, colors or any visual information and they are completely depended on external support which are non-visual methods of interaction with the outer world such as navigation devices, walking sticks, helping partners, gadgets for understanding the surrounding etc. Secondly, Partially Visually Impaired (PVI): These are people with some degree of vision loss, and they still have some remaining vision, they are partially dependent on external help for their day-to-day tasks. PVI individuals suffer from partial loss of peripheral or tunnel vision, due to this they still might be able to differentiate between shades, colors, objects, and depth of field to some extent. This study perceives CVI and PVI individuals based on their ability to operate and use applications or devices without external help. Key components include sound and touch feedback, accessibility, ease of use, voice control, durability, portability, affordability, and compatibility with additional devices. Audio feedback is crucial for conveying device status and environmental information, enhancing user-device interaction. Tactile feedback, through raised buttons or vibrations, enhances user agency and conveys device status.

The current generation has entered the age of smartphones and apps. Due to the proliferation of mobile apps, designers and developers must cater to a wide variety of consumers with specific requirements. Earlier, mobile application designers mainly focused on the features provided. However, nowadays, designers also need to consider the usability, User Interface (UI), User Experience (UX), and ease of use for visually impaired people (7, 8). This is because the earlier Braille system was a good alternative for people who could not use phones, but now, when almost everything requires a smartphone and internet, the need for usable applications for visually impaired people to complete daily tasks easily is inevitable. Many applications have been developed to aid visually impaired people in navigation, item detection, voice commands, etc. These applications still face many open challenges and future directions, which, when addressed, can make the applications better and easier to use for visually impaired people. The authors observed a lack of review papers that comprehensively address the NMUD aspects of both applications and devices for visually impaired users in a single document. The authors realized the need for a detailed review that considers several apps and devices, reviews them based on various parameters, identifies open problems and future challenges, and categorizes them based on their use cases (9).

The existing reviews taken into consideration had several limitations, such as: (a) Some articles did not adequately account for the vast spectrum of differences that exist in the CVI and PVI population. This spectrum encompasses a range of conditions from mild vision loss to total blindness, each with unique challenges and needs. This diversity impacts how individuals interact with technology, necessitating adaptive and inclusive design approaches to cater to the broad array of user preferences and capabilities. (b) In the early 2000s, a lot of attention was not given to the concept of NMUD for the visually impaired since the applications started evolving only after the introduction of smartphones. (c) Many reviews did not consider UI, NUI, UX and Multi-sensory interfaces for their study. (d) None of the studies reviewed both apps and devices together holistically; most studies focused only on applications rather than devices. (e) Overall, there is a limited number of papers and reviews which match this review’s expectations.

**Figure 1:**
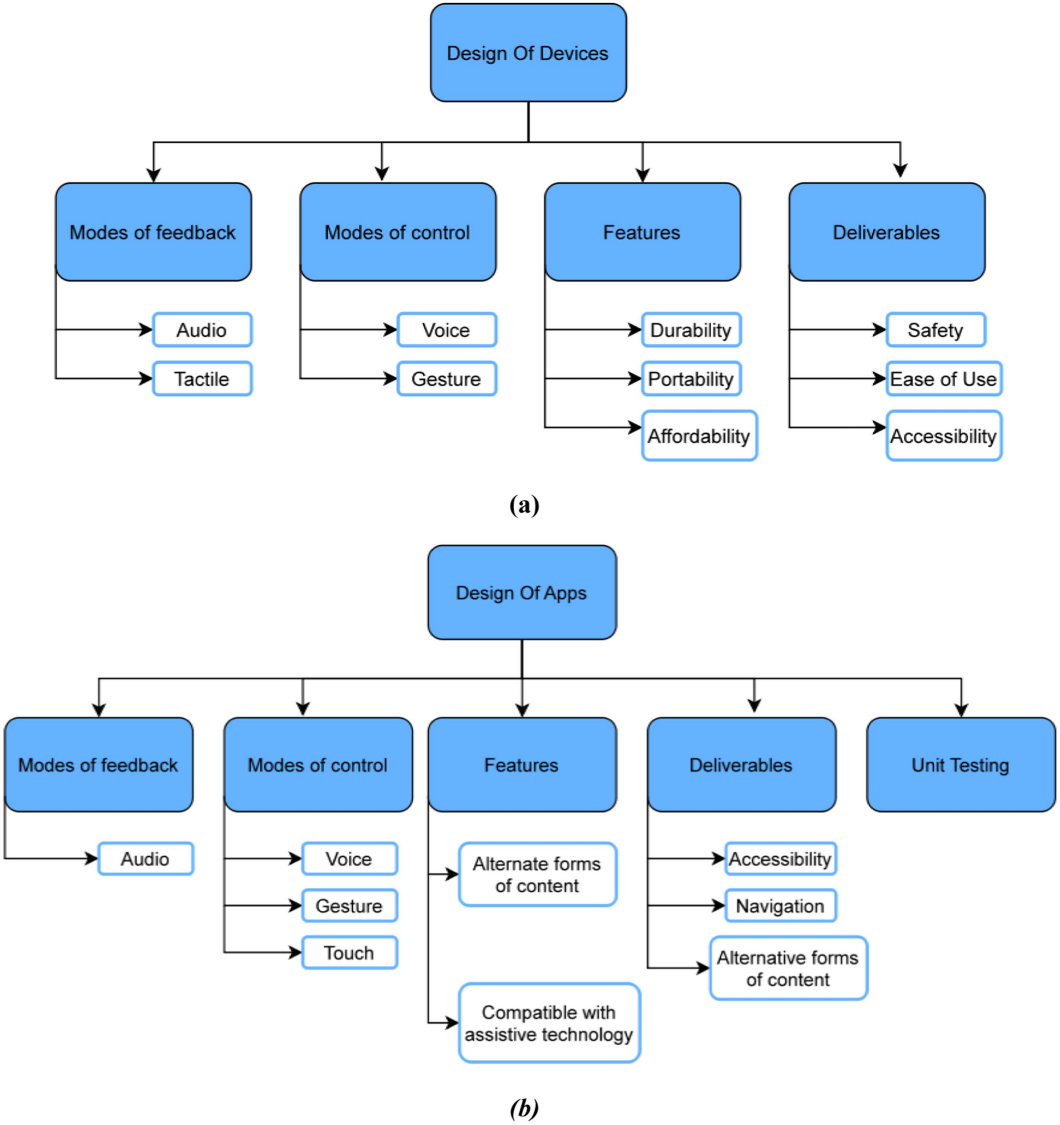
**(a)** Human based elements in the device design for visually impaired; **(b)** human based elements in the app design for visually impaired.

Prioritizing accessibility ensures usability for visually impaired individuals by incorporating adjustable text, larger fonts, increased image sizes, and higher contrast ratios (10). Enhancing user experience involves improving the discoverability and usability of controls. Voice control allows operation without visual cues. Durability determines the longevity and usability of devices for visually impaired individuals, who heavily depend on them. On top of that, portability, defined by weight and size, is essential for the mobility of visually impaired users. Affordability is a critical factor for the success of assistive technologies. Compatibility with other technologies, such as Braille displays and screen readers, facilitates information retrieval and device usage for visually impaired users. Figure 1a represents the human-based elements in device design, detailing features (durability, portability, affordability), deliverables (ease of use, accessibility), modes of control (gestures, voice), and modes of feedback (audio, haptic) (11).

The application’s accessibility should be ensured by following accessibility standards, including high-contrast color schemes, larger font sizes, and adjustable text and image sizes. The navigation design should prioritize intuitiveness and ease of use with clearly labeled buttons and concise instructions. Ensuring compatibility with assistive technologies, such as screen readers and Braille displays, is essential for a comprehensive solution for users with visual impairments. Providing alternative forms of content, such as audio descriptions and audio cues, ensures content accessibility for CVI and PVI users. Figure 1b illustrates the human-based elements in app design for the visually impaired, detailing features (compatibility with assistive technology, smartphone portability), user testing (intense testing with visually impaired users), deliverables (navigation, accessibility), modes of control (gestures, voice, touch), and modes of feedback (audio, haptic via mobile vibrations) (11, 12).

An overview of NUI, Multi-sensory Interfaces and UX Design(NMUD) is also given, wherein discussions about definitions of NMUD for the visually impaired, Human Computer Interaction (HCI), usability, standards and regulations for devices, and methodologies of evaluation are provided (13). It further updates the readers on taxonomy, open challenges, and future research directions after reviewing previously published articles. In the Appendix, Table A1 illustrates the list of abbreviations and definitions. (Appendix Table A1).

### 1.1 Limitations of existing reviews

As observed from the introduction, the authors observed a limited number of review papers that comprehensively address the NMUD aspects of both applications and devices for visually impaired users in a single document. Table 1 presents the comparison with previous review articles based on apps and devices for the visually impaired. It shows that out of the (*n* = 8) review articles (excluding this study) pertaining to applications and devices for the visually impaired, only one article deals with both apps and devices; (*n* = 7) of this concentrate exclusively on apps, and a single article reviews the work done on specialized devices to aid the CVI and PVI. It can also be deduced that although the majority of the articles do address both the drawbacks and future directions, the discussion on future directions lacks clarity and depth (14–22).

**Table 1:** Summary of various devices for visually impaired and their features.

Reference number	Authors and year	Device name	Everyday life use	Information transmitted	How it works	Task	Category
(77)	Kasthuri et al., 2017	Smart Device	No, not commercialized, but proposed to be used as a system, users can tell the device to do something via voice commands - call, play music, fetch live weather reports, transport related information and news updates.	Sound.	Android devices accept voice instructions that are translated into text for immediate execution by Speech Recognition Engine (SRE). The user can retrieve the most recent information via the Selendroid app interface.	Take voice commands, process them and act accordingly.	Communication
(28)	Helal et al., 2001	Drishti	Yes, commercialized, used to help users navigate and reach their destination.	Location, Text, Sound.	Wireless pedestrian navigation system which integrates Geographic information system (GIS) and GPS, and takes various inputs and computes optimized routes and cues for the user.	Returning an optimized route along with cues.	Spatial Orientation and Navigation
(100)	Hoque et al., 2021	Blind Guard	No, not commercialized, to determine barriers.	Touch, Sound	Ultrasonic camera, light and water sensors based blind stick help determine barriers. Application gives option to search object, ask questions to Google, search location, reads document and dials emergency contact number when required.	Search object, ask questions, search location, read document, emergency dial.	Multi-Functional Assistive Technology
(78)	Mon et al., 2021	3D-based haptic, audio, and olfactory-enabled edutainment application	No, not commercialized, can be used to learn shapes.	Touch, Sound, Smell	Blender Software creates three-dimenional (3D) objects and exports them to H3DAPI for haptic and audio rendering, after which features are added to a new Python file for auditory and olfactory feedback.	Learn shapes.	Multi-Functional Assistive Technology
(32)	Huang et al., 2022	SAPSS (Smart Accessible Pedestrian Signal System)	No, not commercialized, can be used to navigate through traffic signals and routes.	Touch, Sound, Signal.	Communication box receives analog signals, makes calculations, and communicates with smartphones through beacons.	Navigate through traffic signals and routes.	Spatial Orientation and Navigation
(29)	Harjumaa et al., 2011	HearMe	Yes, the service is provided via the integrated use of a laptop, NFC card reader and loudspeakers.	Speech output	The text information on the NFC tag attached to the medication package is converted into an audio message using the NFC (Near-Field Communication) technology.	Facilitates to identify the name of the medicine, its dosage and other essential information relating to it.	Healthcare Assistance Technology
(30)	Kim et al., 2013	Smart Cane	Yes, commercialized.	Vibrational alerts using tactile feedback modality, audio signals (tones, speech)	Employs ultrasonic laser or infrared sensors to detect obstacles via touch, echo-sounding, vibrotactile information and produces vibration alerts on detection of obstacles.	Detect obstacles at distances ranging from immediate proximity to greater than 1 m.	Spatial Orientation and Navigation
(79)	Huppert et al., 2021	GuideCopter	No, not commercialized.	Highly precise haptic feedback and audio signals	OptiTracker marker glove uses a well-connected mobile sensor for tracking the user’s hand and responds via multi-modal feedback.	Helps in robust and instinctive localization of objects and everyday routing/traversing tasks.	User Interaction and Environmental Awareness
(108)	Khare et al., 2020	Digi Scribe	No, we need the whole setup.	Voice commands.	Takes voice input, uses STT to get text data, sends text data to scribe, scribe moves pen in defined letter patterns.	Helping CVI users to convert their thoughts in written text.	Communication
(109)	Devi et al., 2020	Smart Navigation Guidance System.	Can be commercialized if all components are packed in a single shell/wearable.	Heart rate, distance, location, image data to the Raspberry Pi.	Various sensors are used to get details about the user, which are then used for aiding users in various ways.	Helps users use a full system of devices for their help.	Healthcare Assistance Technology
(110)	Ehrlich et al., 2017	Head-mounted Display (HMD)	Yes, can be commercialized. It can be used for tasks that require visual enhancement, such as reading, navigating, and possibly even driving	Voice input, trackpad input, eye/head sensing, gaze tracking, hand sensing, and other miscellaneous technologies	They work by presenting enhanced or augmented visual information directly to the user’s eyes. See-through displays superimpose images directly on top of the user’s field of view. Retinal projection devices project images directly onto the retina. Near-eye displays project a see-through image just in front of the eye.	To aid users in low vision rehabilitation and to enhance the vision of individuals with visual impairments. This includes tasks that range from basic daily activities to more complex ones that require precise visual acuity.	Low vision aids (LVAs)

### 1.2 Objectives of this review

- To identify applications and devices that cater to the diverse needs of visually impaired and partially visually impaired users from a NMUD point of view.
- Analyze how well NMUD principles are implemented in these existing solutions and their effectiveness in meeting the users’ needs.
- Identify key NMUD challenges in current solutions and suggest improvements for better usability and user satisfaction.
- Highlight areas for future research to advance NMUD for visually impaired users.

### 1.3 Contribution of this review

Our contribution can be summarized as follows:

- A comprehensive study on the NMUD of apps and devices for the people who suffer from partial or complete visual impairment, including various factors such as everyday life use, information transmitted, working, usability, and universal usability.
- The terms Human-Computer Interaction (HCI), User Interface (UI), UI Design, User Experience (UX), UX Design, Usability, and Universal Usability are defined and explained in relation to user interaction and design. Additionally, a collection of guidelines and standards that govern NMUD for devices and applications designed with the needs of visually impaired individuals in mind is also discussed.
- A comprehensive evaluation of the pros and cons of all apps and devices for the visually impaired, their respective NMUD analysis methods, and the concept of universal usability of user interface design is explored.
- The current open challenges and future research possibilities in the NMUD of apps and devices for the visually impaired useful for aspiring researchers, academia, and industry are presented in this review.

## 2 Methods

### 2.1 Comprehensive review methodology

To systematically choose the articles utilized in this comprehensive review, this study followed the Preferred Reporting Items for Systematic Reviews and Meta-Analyses (PRISMA) technique. This research started with articles (*N* = 3,125) from different article libraries. Out of these, non-duplicate articles (*N* = 2,096) were identified. The below-mentioned inclusion and exclusion criteria were applied, and articles (*N* = 1,859) were removed after the title and abstract screening. The inclusion and exclusion criteria were applied again on the remaining articles (*N* = 237) with full-text screening, and articles not retrieved (*N* = 52). Articles assessed for eligibility (*N* = 185) and (*N* = 79) were eliminated based on the scope of this work. Finally, as a result, (*N* = 106) articles were retrieved to be considered in the study.

These articles (*N* = 106) were analyzed completely by authors Lauryn Arora, Akansh Choudhary, Margi Bhatt and Kathiravan Srinivasan. For each of the articles, the initial step was to identify the future problems and open problems of the article. All the articles taken into consideration were after January 2001 and not before it. Future problems define how the publishers want to scale their products in the future with respect to better user interaction, functionalities, user experience, etc. Open problems define the current issues and/or limitations the publisher’s product is having with respect to user interface, functionalities, user experience. After screening the contents of the articles, the research was broken into different criteria as mentioned in the tables below.

#### 2.1.1 Search strategy and literature sources

**Figure 2:**
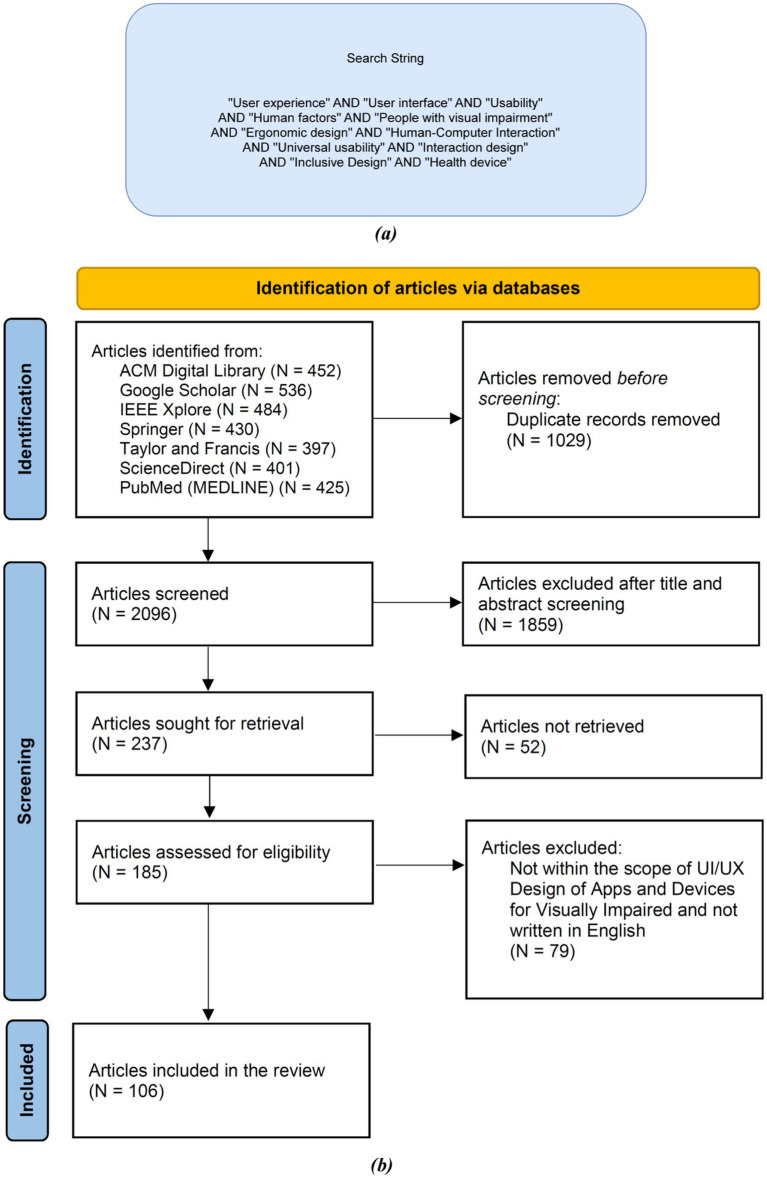
**(a)** Search string used in this review; **(b)** PRISMA flow diagram for the selection process of the research articles used in this review.

For this review, searching for relevant articles was conducted in various databases including Google Scholar, ACM Digital Library, IEEE Xplore, Springer, Taylor & Francis Online, PubMed (MEDLINE) and ScienceDirect from January 2001 to June 2023. Figure 2a illustrates the search string used in this review. A detailed representation of all the major databases considered is provided in the “PRISMA Flow Diagram” (Figure 2b) below. The string elements of this search string were searched on the Google Scholar search engine and the Vellore Institute of Technology ‘e-gateway’ search engine. The search string was formulated after gaining a clear understanding of the keywords that are most relevant to our review. Any article that did not follow the inclusion criteria was not considered. Both the inclusion and exclusion criteria are explained below.

#### 2.1.2 Inclusion criteria

The inclusion criteria for the articles in this review are as follows: First, the articles must be published after the year 2001. Second, they must be related to aiding the CVI and PVI individuals. Third, the articles can contain prototypes, concepts, or full projects/applications. Fourth, they must be written entirely in English. Fifth, they should be published in peer reviewed journals. Sixth, the articles should be either review articles or research articles. Finally, connection to the user interface (UI) and user experience (UX) design of apps or devices for visually impaired is required.

#### 2.1.3 Exclusion criteria

The exclusion criteria for the articles in this review are as follows: First, articles should not be grey articles and published in archives will be excluded. Second, articles that have very little focus on aiding the CVI and PVI will also be eliminated. Third, articles that do not have a clear goal on the product will not be considered. Fourth, articles that are solely based on opinions rather than empirical evidence will be excluded. Fifth, conference abstracts will not be included in the review. Sixth, case reports or case series will also be eliminated from the review. Finally, letters to the editor will not be considered as part of this review.

#### 2.1.4 Risk of bias

Selection of studies was made using PRISMA 2020 guidelines in this comprehensive review, which is an unbiased search strategy, along with detailed inclusion and exclusion criteria defined in sections 2.1.2 and 2.1.3, respectively. These were used to find full-length peer-reviewed articles. Efforts were made to minimize bias in selection based on the inclusion criteria, exclusion criteria, PRISMA 2020 guidelines, and search string. The focus has been on reporting studies with positive and statistically significant data from well-known journals mentioned in Figure 2b. PRISMA 2020 guidelines were employed to exclude grey and unpublished articles and literature, thus significantly reducing publication bias. Only peer-reviewed journals were considered to maintain the authenticity of the publications. There is a possibility of language bias, as only articles and studies written in English were considered for this review. This was due to English being the common language understood by all the authors and individuals involved in the review. The data presented in this review is based on existing peer-reviewed articles, with data reported directly from the referenced works. Best efforts have been made to obtain valid data from these peer-reviewed articles to curtail reporting bias as much as possible.

#### 2.1.5 Safety concerns

After carefully observing and reading the details on the applications and devices mentioned in the review, the authors noticed several safety concerns evaluated based on how it will impact a visually impaired person’s daily routine and personal information:

- Probability of data leaks when audio and sensory applications record user data and store it in their databases.
- Probability of accidents of mishaps when the devices are being worn around the body and the person is still in learning stages.
- There will be an increased level of fear and anxiety since now the person has to put all their trust regarding navigation and information on an electronic device which can function in unexpected ways due to technical glitches.

## 3 Results

To guarantee the inclusion of pertinent and excellent research, we conducted a thorough study selection process as the first stage of our analysis. Starting with nearly 3,125 results; 106 papers that satisfied the inclusion and exclusion criteria were found following a thorough screening procedure. These studies were assessed according to how well they adhered to NMUD principles and how applicable they were in real life to help the visually impaired population. Methodological rigor, publication year, peer-reviewed status and several other aspects were used to evaluate the research’ quality. This resulted in a collection of prototypes, concepts, and fully functional apps and devices/gadgets which provides a wide view of the current conditions and ongoing difficulties in the field of NMUD for the visually impaired population.

**Figure 3:**
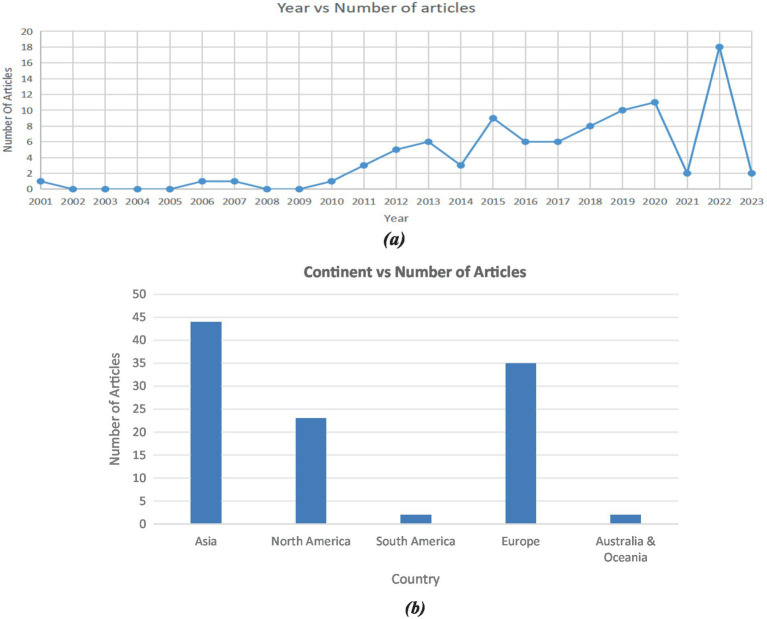
**(a)** Trend of studies on NMUD design of apps and devices for visually impaired over time; **(b)** trend of studies on NMUD design of apps and devices for visually impaired by continent.

After implementing the comprehensive review methodology, inclusion criteria, exclusion criteria, and search strategy on all the literature sources, we constructed the “PRISMA Flow Diagram” (Figure 2b) which explains the filtration process applied to articles, research articles, and various different types of studies, using a defined search string and inclusion and exclusion criteria, to get to the final result set. “Article Count vs. Year” (Figure 3a) demonstrates the comparison between year and number of articles, which helps us understand how researched the topic was in different years. The graph trend shows that researchers are more inclined toward helping the visually impaired in recent years compared to before. These illustrations help us better understand statistics on the literature sources that were collected for this review. Figure 3b illustrates the trend of studies on NMUD of Apps and Devices for the Visually Impaired by country. It helps us understand how researched the topic is in different countries, and the graph trend shows that researchers are more inclined toward helping the visually impaired in India (*N* = 14) and the United States (*N* = 8).

### 3.1 Apps and devices for visual impairment

#### 3.1.1 Mode of action for people with visual impairment

People with visual impairment may use a variety of strategies and technologies to navigate their surroundings and access information. Some common modes of action are:

- Auditory cues: Auditory cues have high potential for improving the quality of life of people who are visually impaired by increasing their mobility range and independence level (23).
- Tactile cues: Touch or tactile cues are used to give the individual with dual sensory impairments a way of understanding about activities, people and places through the use of touch and/or movement (24, 25). For instance, they might use their sense of touch to distinguish between various textures or surfaces, such as using a cane to detect changes in the ground surface.
- Assistive technologies: Braille displays, magnifying software, and screen readers, white canes are included (26). These tools facilitate easier interaction for people with vision impairment with others online and access to digital information.
- Mobility and orientation instruction: People with vision impairment receive instruction in safe and independent mobility. Orientation and mobility (O&M) instruction provides students who are deaf-blind with a set of foundational skills to use residual visual, auditory, and other sensory information to understand their environments (27).

Support from friends, family, and professionals is important for many people with vision impairment.

#### 3.1.2 Devices for visually impaired people

Table 1 analyses research articles on devices for the visually challenged, highlighting important aspects such as daily use, information transmission, functionality, and tasks. It can be inferred that just 4 out of the 10 proposed solutions that we examine here have been commercialised and are in actual use by the target audience (28–31). In most devices, the information is communicated to the users via sound and, in some cases, via haptic feedback. In a few cases, the devices store and transmit geographical data, such as images of the surroundings and distance-related data. The solutions employ motion, sound, and obstacle detection sensors, and Global Positioning System (GPS) for their operations (28, 30, 32); technologies like Near-Field Communication (NFC) tags have also been seen to be applied. These devices can assist users with a wide range of tasks, from learning-related tasks to navigation and obstacle detection (33–36).

#### 3.1.3 Apps for visually impaired people

Table 2 reviews research articles (*n* = 43) on apps for the visually challenged, focus on crucial aspects such as daily use, information transmission, functionality, and tasks. It can be inferred that (*n* = 24) apps out of the proposed solutions that we examine here have not been commercialised, whereas (*n* = 19) of these applications are in actual use by CVI and PVI users. In the majority of applications, the information is communicated to the users via text, sounds, audio, and, in some cases, Braille text and haptic feedback. In a few cases, the applications store and transmit geographical data, such as images of the surroundings and distance-related data. The solutions employ motion, sound, and obstacle detection sensors, cameras, and GPS for their operations (32, 36–71). Most of these applications aim to make a regular smartphone more accessible to CVI and PVI users and utilize the wide range of functionalities that make their lives simpler and assist them in learning, navigation, shopping, traveling, and entertainment-related tasks.

**Table 2:** Summary of various apps for visually impaired and their features.

Reference number	Authors and year	App name	Everyday life use	Information transmitted	How it works	Task	Category
(37)	Chen et al., 2015	BlindNavi	No, not commercialized, can be used as an android application.	Auditory, olfactory, or tactile clues	A three-step, flat-low design, voice feedback with multisensory signals, micro location technology, and the provision of multisensory messaging combining well-known reference locations that users had learnt from O&M training are all incorporated.	Provide navigation clues to the user by providing multi-sensory cues.	Spatial Orientation and Navigation
(111)	Sierra et al., 2012	Low Vision Mobile App Portal	No, not commercialized.	Collection of apps on portal	Use of low vision wheel control and low vision segmented control in low vision library to use controls in the apps on portal, which guide users to perform various tasks.	Calling, messaging, putting an alarm, checking battery, checking emails, using magnifier, checking GPS Location	Communication and Accessibility Tools
(112)	Liu et al., 2015	Voice Helper	No, not commercialized.	Audio, Text	Reader converts text-to-speech (TTS) and helps CVI and PVI persons for operating devices. Message reader, Optical Character Recognition (OCR) reader, text file reader, and voice dialing is also integrated in Voice Helper. Navigation Reader is also integrated for destination distance and moving directions.	Message reading, Text file reading, OCR reading, voice dialing and navigating users.	Multi-Functional Assistive Technology
(99)	Zhang et al.,2021	Voicemoji	No, not commercialized, can be used as a web application.	Text, Emoji	Voice input is parsed using speech-to-text Application Programming Interface (API), emoji is searched using the text, emojis are modified if command is given. Emoji suggestions are given based on context provided by voice command.	Emoji searching, entering text and emoji via voice command, using suggestions provided by voice command context if needed.	Communication and Accessibility Tools
(93)	Nahar et al., 2015	mBRAILLE	No, not commercialized, proposed to be implemented as an android application.	Text	Six logical gestural switches used for learning alphabets with the help of a basic Braille system.	Learning letters and words in Bangla and English using Braille.	Education
(113)	Eduardo et al., 2016	All Appointments	No, not commercialized, proposed to be implemented as an android application.	Audio	Voice commands get translated via Speech-to-text API and parsed, a Google search on emoji descriptions is done and relevant emoji is inserted into the text.	Manage appointments by use of voice commands.	Communication
(94)	Barata et al., 2018	Intelligent Software Assistant	No, not commercialized, can be used as an android application.	Audio, Text	Speech-to-text feature allows the user to give voice commands which are then performed by the system. Incoming message sender’s name and content are read out by text-to-speech functionality.	Calling and messaging.	Communication and Accessibility Tools
(39)	Landazabal et al., 2019	TransmiGuia	No, not commercialized, can be used as an application.	Audio, Location	People who are visually challenged can utilize speech recognition and geolocation to navigate the public transportation system.	Reach the nearest stations.	Spatial Orientation and Navigation
(90)	Marvin., 2020	DAVID	No, not commercialized, can be used as an android application.	Audio, Text	The Google Cloud Speech to Text API is used to convert audio to text. The central processor receives text and executes the assigned task. In order to find text in photos and video streams, one must use the Google Mobile Vision Text Recognition API. The Google Cloud Text to Speech API, which produces voice output, is utilized to read the text.	Knowing time, knowing nearest bus stations, reading text from an image or a video.	Communication and Accessibility Tools
(96)	Yousef et al., 2013	Mobile Phone Dialler Application	No, not commercialized, can be used as a windows application.	Touch, Nodes	6 nodes are displayed on screen, Nodes-based dialler (NBD) algorithm was called and then call was made.	Calling.	Multi-Functional Assistive Technology
(100)	Hoque et al., 2021	Blind Guard	No, not commercialized, proposed to be implemented as an android application.	Touch, Sound	Ultrasonic camera, light and water sensors based blind stick helps determine barriers. Application gives option to search object, ask questions to Google, search location, reads document and dials emergency contact number when required.	Search object, ask questions, search location, read document, emergency dial.	Multi-Functional Assistive Technology
(78)	Mon et al., 2021	3DOHA application	No, not commercialized, can be used as an application.	Touch, Sound, Smell	Blender Software creates a 3D object, which is exported to H3DAPI, where haptic and audio rendering is done. After that, a Python file is created and features are added, where auditory and olfactory feedback is given.	Learn shapes	Multi-Functional Assistive Technology
(32)	Huang et al., 2022	SAPSS (Smart Accessible Pedestrian Signal System)	No, not commercialized, can be used as an android and iOS application.	Touch, Sound, Signal.	After downloading the program, users may accurately geo-locate themselves using their cell phones to get pedestrian signals in real-time, and the SAPSS box can communicate with them via Bluetooth.	Navigate through traffic signals and routes.	Spatial Orientation and Navigation
(101)	Gooda Sahib et al., 2015	TrailNote	Yes, commercialized.	Audio(speech and non-speech sounds)	The interaction with the application is done with the help of a speech-based screen reader.	This application aids the CVI and PVI users to refine and enhance the searching process and also comes with a note-taking feature.	Communication and Accessibility Tools
(102)	Anagnostakis et al., 2016	Prototype system for Accessible Museum Collections	No, not commercialized,can be used as a mobile application.	Tactile, audio	The user selects a topical area and a display piece and the technology is able to direct you to your destination, enabling the user to tactually explore (using capacitive sensors) the exhibit supplemented with audio descriptions.	An audio description is generated along as one touches the statue giving an interactive experience and realizing their form and structure.	Sensory Enhancement Technology
(103)	Chippendale et al., 2015	Personal Shopping Assistance and Navigator System for Visually Impaired People	No, not commercialized, proposed to be implemented as an android application,	Audio, haptic	The system interacts with the user by using Augmented Reality. Specific objects are detected and recognized and the system responds to the user via multi-modalities.	This application helps the user to navigate and acts as a personal shopping assistant providing a very stimulating experience.	Commerce and Accessibility Tools
(88)	Stein et al., 2011	Auditory vocabulary and spelling trainer (AVoS)	Yes, commercialized.	Audio	It combines braille and text-to-speech technology together, that is, the system is controlled using both screen readers and the user interprets the output using a braille display.	E-learning device that functions as an auditory vocabulary and spelling trainer.	Education
(91)	Cahya et al., 2018	MBT using Double Diamond Approach	Yes, can be installed in a smartphone device.	Voice, braille letters	Dynamic virtual braille keyboard with a swipe screen and 6 braille dot buttons act as input sources and output is generated via voice commands and braille text view.	Serves as a self-educate application with functionalities to learn, write, exercise, translate etc	Education
(49)	Álvarez Robles et al., 2019	Mobile Geolocation system	Yes, commercialized and installed in an android device.	Audio	Using a programmed API, the system recognizes the speech order and requests for the location of the GPS sensor, then a text-to-speech API is called responding to the user through audio feedback.	Gives the freedom to CVI and PVI users to independently move around and interconnect better with their environment.	Spatial Orientation and Navigation
(92)	Wang et al., 2019	EarTouch	Yes, commercialized.	Speech	Enables single hand holding of the device to give command/request via touch gesture using ear, the system detects and responds via speech output privately.	Facilitates the users to interact with the application by performing gestures using the ear.	Communication and Accessibility Tools
(95)	Sagale et al.,2018	Eye-Free Android Application	Not commercialized, a prototype based on existing commercialized applications.	Braille text, speech.	Two modules: Sending module - A speech input is converted to text using an STT converter and the text invokes a specific function. Receiving Module- The response /action performed by the system is converted to text which is further converted to a speech output by the user using TTS converter.	Provides users with basic calling and messaging functions, can be extended to provide functionalities like multimedia, gaming and navigation support.	Multi-Functional Assistive Technology
(89)	Russ., 2017	i-AIM App	Yes, commercialized.	Audio, vibrational signals	By fusing the i-AIM app with Hololens/Lite-glasses, we can generate an aural representation of space within the Single in-depth vertical application (sounds).	Enables the user to detect and determine objects and the distance and direction they are placed in.	Sensory Enhancement Technology
(105)	Kangeswaran et al., 2021	Bilingual Audio Based Online Shopping Mobile Application	No, not commercialized.	Audio	The application has both a graphical and voice-based user interface and uses Natural Language Processing (NLP). The user can select a language for both the directions and navigation tasks within an application.	Assists in online shopping and provides a smooth and immersive experience to CVI and PVI users.	Commerce and Accessibility Tools
(59)	Rizan et al., 2021	Guided Vision	No, not commercialized.	Audio	The application is connected to ESP32CAM(connected through Bluetooth) and remote server simultaneously. Obstacles are detected instantaneously by stationing a deep-learning model in the system.	Skills in reading, identification, description, and safe navigation	Spatial Orientation and Navigation
(114)	Jakhete et al., 2019	Object Recognition App	No, not commercialized but can be used via a hand-held device like a mobile phone.	Audio	For both object recognition and detection, the SSD (Single Shot Detector) Algorithm is utilized. In addition, this algorithm provides nearly accurate results for real-time object detection and has been shown to be speedier than comparable algorithms. In addition, the application employs android TensorFlow Protocols and android Text-to-speech API for audible output.	Identifying and finding objects.	Multi-Functional Assistive Technology
(100)	Hoque et al., 2021	Blind Guard	No, not commercialized.	Audio (alert sounds and speech), location	A photo is selected by the user. The circuit will not activate if the barrier is too far away. A microcontroller can transmit a signal to activate a buzzer if the barrier is getting too close. A second buzzer is sounded to inform the CVI of the presence of water.	Provide the CVI individual with direction, protection, and safety. It would be simpler for blind individuals to navigate and communicate with others. They will quickly find anything they need.	Multi-Functional Assistive Technology
(97)	Robles et al., 2019	Talkback	Yes, commercialized.	Organized menu utilizing the semi-closed card-sorting approach	The precise arrangement and selection of menu items in a way that is intuitive for visually impaired users is defined.	An accessibility feature enables them to move around the various applications and tools on the device.	User Interface and Accessibility Enhancement
(20)	Buzzi et al., 2018	Simple Smart Homes Web Interface for Completely Visually Impaired People	Yes, can be commercialized.	Internet of Things (IOT) devices spread across the home.	Buttons and interfaces given to users to easily navigate and change various home elements.	Interactive elements provided to users to use IOT enabled home devices.	Multi-Functional Assistive Technology
(115)	Ariane et al., 2021	An Image Captioner	Yes, can be made into Mobile application	Surrounding images passed.	Neural Network created for image understand and captioning	To take image input from user and process it into a text description	Multi-Functional Assistive Technology
(106)	Senarathne et al., 2021	BlindAid	Yes, can be made into Mobile application	Ultrasonic sensor and camera.	BlindAid is capable of reading signs and labels, recognizing faces, identifying emotions, measuring distances to identify obstacles, and more.	BlindAid is capable of recognizing faces, recognizing emotions, measuring distances to differentiate between barriers and objects, and extracting data from bills, posters, and product labels.	Multi-Functional Assistive Technology
(31)	De Silva et al., 2017	iShop	Yes, can be made into Mobile application	Predefined map of the shop and users location.	The application determines the shelf order to travel in, the number of steps to be taken, the turns to make, and the instructions to each item. It then narrates each step to the user one at a time. Utilizing a step counter sensor, it tracks the user’s steps and, once the target is reached, switches the directions on its own.	IShop’s primary goal is to improve and streamline the shopping experience for persons who are CVI or PVI	Commerce and Accessibility Tools
(116)	Usharani et al., 2020	Voice Based Form Filling System	Yes, can be made into Mobile application	Headset button and user’s voice	Using various interactive methods, users are able to fill form fields one by one via speech commands from opening the app to submitting the form.	Help users in Filling bank forms via speech input.	Communication and Accessibility Tools
(117)	Calabrò et al., 2010	Book4All	No, it needs a computer for running the prototype.	PDF extractor tools and parsing tools.	Analyses the data that was taken from the e-book to reconstruct its semantic structure, then stores the results in the Intermediate Book Format.	A PDF e-book may be converted using the suggested program into a more acceptable, usable, and accessible version that can be read on a desktop computer or on a mobile device.	Education
(118)	Nayak et al., 2020	Assistive Mobile Application for Visually Impaired People	Yes, can be made into Mobile application	Speech input.	The CVI (blind people) can produce PDF files, send emails to predetermined addresses, and even hear transaction-related Short Message Service (SMS). This application uses the calendar API to manage events for those who are vision challenged. With the aid of the GPS-based navigation offered by this program, blind people may travel by foot and arrive at their destination.	Convert user speech commands into actions and help them send messages, GPS, schedule tasks.	Multi-Functional Assistive Technology
(98)	Frey et al., 2011	BrailleTouch	Yes, it can be built into smartphones.	User touch patterns on the screen.	Users hold the BrailleTouch device in two hands and use it with their fingers in a layout and manner that is identical to that of a Braille writer. The left index finger covers key 1, the left middle finger covers key 2, and so on, to put it simply. The mental diagram of the six cells of a Braille character corresponds geographically to the six buttons on BrailleTouch.	To convert the user touch pattern to braille letters.	Sensory Enhancement Technology
(62)	Harrington et al., 2012	ABLE Transit	No, just a prototype for now.	Users’ GPS location.	This experiment uses iPhone plugins to access a SQLite database and obtain General Transit Format Specification (GTFS) data. The database’s schema and the GTFS data’s schema are same. Using the user’s current GPS coordinates, the program downloads the GTFS data, loads it into a database, then queries the database to find the closest bus stop.	To help visually impaired users near bus stops to gain bus information and arrival times.	Spatial Orientation and Navigation
(107)	Ceccarini et al., 2019	Tourism for all	Yes, Commercialized as a mobile app.	Pre-fed tourism data along with user demographics and location.	Application built and developed to fulfill the needs of users with visual impairments, including poor vision and CVI users, and to provide an accessible marketplace where associations/organizations and private businesses may provide their tourism-related services. The system used gamification and suggested strategy to engage and encourage people to utilize the system.	Help CVI people enjoy tourist services.	Multi-Functional Assistive Technology
(119)	Bagwan et al., 2015	VisualPal	Yes, mobile application ready to use.	Images clicked by the user.	Application takes in image input from the user and then uses the custom made object and color recognition model to understand elements of the image and speak out loud a description of the image.	Helps people listen about their surroundings by just taking pictures.	Communication and Accessibility Tools
92	Shen et al., 2012	Real time OCR application.	No, only a proposed prototype.	Phone’s camera.	Takes in images from users, does OCR analysis and gives speech output using TTS.	Helps users understand text and words near them using a phone camera.	Sensory Enhancement Technology
(65)	Lin et al., 2014	Safewalking Application	Yes, Android App	Crowd sourced data stored and analyzed on the cloud.	Data about roads and staircases in between are taken from non-CVI and non-PVI users’ phones and is used to alert visually impaired people when they traverse that same path.	To help CVI and PVI people avoid falling off stairs in their pathway.	Spatial Orientation and Navigation
(120)	Akkapusit et al., 2021	Task-Oriented Approach to Guiding People with Visual Impairments.	Not available commercially.	User’s voice command and finger location tracking.	User tells a talk via voice and the phone verbally guides the user’s finger over the screen to help them click the right buttons.	Help users use smartphones with the help of voice guidance.	Communication and Accessibility Tools
(67)	Suresh et al., 2020	Vision	Yes, Android application	Users voice and location.	Takes in user’s voice input and location input to perform various tasks like Google Maps lookup, Navigation, News Reader, Remainder keeper and provide voice output for each.	Allows users to have an easy day to day life by providing several functionalities in one app.	Multi-Functional Assistive Technology
(68)	Chatzina et al., 2021	Route Planning and Navigation Aid App	No, just a presented framework	Crowdsourced data about issues on roads and user’s location	Takes in crowdsourced data about potholes, road quality, safety etc. and then helps CVI users navigate via the safest route.	Helps users navigate via a safe route avoiding potholes and unsafe areas.	Spatial Orientation and Navigation
(121)	Lin et al., 2017	Simple Smartphone-Based Guiding Application	Yes, not commercialized	Audio, Haptic feedback, User Location	Combines real-time audio and haptic feedback to assist visually impaired users in navigation and daily tasks	Navigation, Daily Tasks	Spatial Orientation and Navigation

### 3.2 NMUD design

#### 3.2.1 Human–computer interaction

Human-computer interaction is a cross-disciplinary area of study, which majorly focuses on how to build, develop, and evaluate systems based on design thinking and implementation that emphasizes the communication between users and devices. The HCI system comprises four crucial elements: user, task, tool, and context (72). Since HCI takes into account human behavior, it is closely linked with fields like cognitive psychology, sociology, anthropology, and cognitive science. HCI’s close relationship with multiple disciplines of human behavior as well as computer science has made it an ever-evolving field, and its evolution has been influenced by the latest technological advancements (73–75).

#### 3.2.2 User interface and user interface design

The most common are graphical user interfaces, interfaces controlled by voice, and interfaces based on hand gestures (76). However, User Interface design refers to a careful process of designing and developing these interfaces that lead to a seamless experience for the users. A very important element is that this design must be “invisible,” meaning while the user interacts with this system his attention must not be utilized in the design itself but rather support him in the quick, easy, and effective implementation of the required tasks (6, 76).

#### 3.2.3 UX and user experience design

UX design requires the knowledge of programming concepts, cognitive science, interactive designing, graphic and visual designing. Table 3 presents the user comments on User Feedback - NMUD, Usability, and Universal Usability for apps used by Visually Impaired. It provides a comprehensive assessment of research publications (*n* = 43) pertaining to applications designed for those with visual impairments. The primary focus of this review is on key factors like usability, universal usability, and universal feedback. Moreover, a significant proportion of scholarly articles (*n* = 25) fail to address user feedback, while the remaining articles include user perspectives by highlighting the most beneficial elements, proposing novel functionalities, and suggesting modifications. Table 4 provides the definitions of User Feedback—NMUD, Usability, and Universal Usability for devices used by Visually Impaired. It reviews research articles (*n* = 10) on devices for the CVI and PVI individuals, focusing on crucial aspects such as usability, universal usability, and universal feedback. The majority of user comments on the usability have been that the application was found easy to use and successful in making their day-to-day tasks more convenient. It can be deduced that only five of the apps offer universal usability (29, 30, 77–79); and the rest do not, largely due to language constraints. Furthermore, three articles do not discuss user feedback at all, and the comments for the rest include the most useful features according to users, as well as recommendations on new features and suggested changes.

**Table 3:** Definitions of user feedback—NMUD, usability and universal usability for apps used by visually impaired.

Reference number	Authors and year	Target app for the visually impaired	Usability	Universal usability	User feedback—NMUD design
(37)	Chen et al., 2015	BlindNavi (Navigation Application)	Easy and convenient to use, but needs some changes as per user feedback.	Yes	Bus dynamic system, search History memorization, save favorite addresses, emergency call button, location reuse, search points of interests, landmarks category features were found desirable to be added. “Public transportation around” and “Where am I?” functions to be repositioned.
(111)	Sierra et al., 2012	Low Vision Wheel & Segmented Control	N/A	Yes	N/A
(112)	Liu et al., 2015	Voice Helper (Personal Digital Assistant)	N/A	N/A	N/A
(99)	Zhang et al., 2021	Voicemoji (Keyboard-based emoji entry)	Practical and easy to use, emojis can be easily entered, recommendations are offered, emoji research is encouraged, and communication is improved.	Yes	Emojis can be easily entered, suggestions are useful, emoji exploration is supported, and enriching communication, cumbersome to switch as it is a web app, it should be made to an actual keyboard.
(93)	Nahar et al., 2015	mBraille (Braille Learning Application)	Easy to distinguish user interface layout, no prior knowledge of Braille required, clear instructions, option to correct mistakes, get error messages when wrong step taken and how to correct it.	No	Cheap, excellent idea and good option of Bangla apart from English
(113)	Ghidini, et al., 2016	All Appointments (Appointment manager)	Easier event creation procedure, intuitive and practical use.	No, only English and Spanish are available	Removing the requirement to click the system’s message button and the confirmation prompt when typing, as well as improving the acoustic feedback on the keyboard; adding a text field for random appointment data; illustrative voice commands for beginners; having a date field’s year automatically filled in; navigated between entries on a paginated list and described the current appointment.
(94)	Barata et al., 2018	Intelligent Software Assistant (Personal Digital Assistant)	N/A	Yes, English and Indonesian	N/A
(39)	Landazabal et al., 2019	TransmiGuia (Public transportation services assistant)	Takes into account web delays, provides users with estimated distance and time to their destination, shows nearby stops according to the user’s current location and accidents/traffic jams information.	No	N/A
(90)	Marvin., 2020	DAVID	Able to interpret user speech input with ease and carry out the user’s request	Yes	N/A
(96)	Yousef et al., 2013	Mobile Phone Dialler Application	Several speech recognition issues are resolved, and it is simple to use. Increased consumers’ independence and stress tolerance when dialing phone numbers.	No	It is practical, simple to use and understand, gives users confidence that they are dialing the right number, runs the program faster than speech recognition software, and makes using a mobile phone more autonomous and less stressful.
(100)	Hoque et al., 2021	Blind Guard	N/A	Yes, in English and will be in Bangla	N/A
(78)	Mon et al., 2021	3DOHA application	Efficient, simple to use, and delighted with the program.	No	Efficient, simple to use, and delighted with the program.
(32)	Huang et al., 2022	SAPSS (Smart Accessible Pedestrian Signal System)	Interface with a minimal design that is simple to learn and remember; SAPSS transmits voice message to user’s mobile phone near control box; audio cues optimized for easier use; app’s volume limit set at a permissible extreme; extra sound beams transmitted from across the road.	No, only in Chinese	Experiences that are instructive, memorable, successful, and fulfilling.
(101)	Gooda Sahib et al., 2015	Web searching Interface	New features such as “Tag as Useful” and spelling suggestions were found useful and the overall response was positive with a few minor suggestions.	Yes, the interface is in English which can be accessed by most people across the globe.	Found the interface to be simple, accessible, orderly and precise
(102)	Anagnostakis et al., 2016	Application for accessing Museum Collections	Helps users get about the museum and get a feel for the exhibits.	No, due to language constraints.	This system has unlocked a whole new dimension for museum-going, making it simple to control audio descriptions using gestures and providing a pleasant experience for the visitor.
(103)	Chippendale et al., 2015	Shopping and navigation assistant android application (uses a smartphone connected to a smartwatch)	Although highly functional, complicated to understand, has a low latency, requires to change camera orientations	No, due to language constraints.	Require a tutorial to learn the system prior to first use, low system latency
(88)	Stein et al., 2011	Auditory vocabulary and spelling trainer (AVoS)	Promising response owing to the features of orthographic feedback and a user friendly screen reader with the context of the word.	No, due to language constraints.	Many features like pronunciation help, orthographic feedback was found to be desirable
(91)	Cahya et al., 2018	Braille learning Application	Replacement of the static keyboard with a dynamic keyboard with dot braille and addition of a swipe writing assisted button based on user needs.	Yes, touch based input and feedback is in the form of braille output/speech (Google’s text to speech API).	N/A (User feedback was considered prior to development).
(49)	Robles et al., 2019	Mobile Geolocation system	The system lacks internal locus of control, low latency and not confidential	No, due to language constraints.	Delayed and inadequate feedback found to be distressful, scared of getting lost, family members found it to be beneficial.
(92)	Wang et al., 2019	Smartphone interaction technique	Efficient, easy, fun and socially acceptable	Input methodology is universally acceptable but output audio has to be language specific,	Easy to learn, found the technique to be satisfactory to fulfill their daily needs.
(95)	Sagale et al., 2018	Multi-purpose eye-free android application	Multi-functional, adequate multimodal feedback, needful action confirmation.	Regional and foreign languages have not been incorporated yet.	N/A
(89)	Russ., 2017	Navigation assistant	Accessible provides customized information, Highly usable, can be personalized	No, audio descriptions required for each language	N/A
(105)	Kangeswaran et al., 2021	Online Shopping Mobile Application	Gives users freedom in a convenient manner performing all operations using voice recognition.	No (only available in English and Tamil)	N/A
(59)	Rizan et al., 2021	Guided Vision (Navigation Support Application)	Identifies humans, detects obstacles and describes them in a highly efficient and low latent manner.	No, audio descriptions will require different languages.	N/A
(114)	Jakhete et al., 2019	Object Recognition Application	Recognizes objects and gives an audio output in real time. Accurate and quick response	No, audio descriptions will require different languages.	N/A
(100)	Hoque eta l., 2021	Communication and navigation support application	Gives exact results, error free, multi-functional	No, all the features are available only in Bangla language.	Acceptable and error free
(97)	Robles et al., 2019	Talkback	Describes exactly the intuitive ordering and choices of menu items that are appropriate for CVI and PVI users.	Yes, a generalized approach that can be adapted to any version of android devices	Reliable, intuitive and friendly
(20)	Buzzi et al., 2018	Personal Digital Assistant	Easily understand user button input and accordingly turn on/off home appliances	No (Only in English)	Test subjects found the app easy to use and helpful.
(115)	Ariane et al., 2021	Image Captioner(Mobile application)	Easily convert photos into text captions	No (Only in English)	N/A
(106)	Senarathne et all., 2021	BlindAid(Personal Digital Assistant)	Perform face and emotion recognition, distinguish barriers from objects using distance measurement, and extract data from money, product, and sign board labels.	No (Only in English)	N/A
(31)	De Silva et al., 2017	iShop(Personal Shopping Assistant)	Management of shopping lists, navigation, location-based advertising, product recognition, immediate assistance, inventory management, and customization of store plans.	No(Only in English)	N/A
(116)	Usharani et al., 2020	Voice based form filling- Personal Digital Assistant	Easily fills in details from the voice input of the user.	English and Regional Language Support	N/A
(117)	Calabrò et al., 2010	Desktop application - Educational Digital tool	Aimed at resolving the problems with usability and accessibility in electronic publication.	No (Only English)	N/A
(118)	Sriraksha Nayak, Chandrakala C. B.	Personal Assistive Mobile Application	Speak the feature you want and follow the system’s voice instructions to get there.	English, Hindi, and Kannada	N/A
(98)	Frey et al., 2011	BrailleTouch: Personal Messaging Assistant	An eyes-free text entry technology for touch screens.	Yes, available in various languages	Two braille teachers who examined the app agreed that it has a lot of potential in this field. We learned from our initial interviews that they do not read Braille with their thumbs. Because it is too thick, the thumb’s skin cannot be delicate enough to read.
(62)	Harrington et al., 2012	ABLE Transit: Personal Digital Assistant	Accessible Bussing via Location Estimation (ABLE) Transit provides customers with bus schedule information while requesting the least amount of personal information possible.	No, English Only	N/A
(107)	Ceccarini et al., 2019	Tourist service aid assistant	Permit access to facts on the destination’s accessible services and activities, including specifics about personal support services and accessible transportation options.	No, Italian Only	The main issue was with the sharing services option, which was seen to be really fascinating but not really simple to discover.
(119)	Bagwan et al., 2015	Visual Pal: Personal Digital Assistant	Easily capture images and give image/object recognition with audio output.	No, English Only	N/A
(122)	Shen et al., 2012	Personal Digital Assistant	An innovative audio-tactile user interface lets the user hold the smartphone level and helps him or her locate any relevant text and, if required, approach it for a sharper view.	No, Can only detect English	Two participants were chosen for the testing process. Finding text in an unfamiliar environment was the system’s main problem, which required the user to carefully examine wide regions. Motion blur and poor processing rates made it necessary for the user to scan slowly.
(65)	Lin et al., 2014	Personal safe navigation assistant	Special topographic map specific for the visually impaired people which contains information about staircases, potholes provided by crowd-sourcing	No, English Only	N/A
(120)	Akkapusit et al., 2021	Personal Digital Assistant	Adaptive guidance is provided by a novel object identification technique used by a guidance system to identify smart device control panels and identifies the user’s intended job.	No	Participants said that when compared to the other two activities, the button was too tiny to accurately detect where to touch, and the voice assistant occasionally misrepresented the button.
(67)	Suresh et al., 2020	Vision: Personal Digital Assistant	Easy Object Detection, Google Map Navigation, Rss News Reader, Alarm Keeper.	No, Only English	N/A
(68)	Chatzina et al., 2021	Route Planning and Navigation Aid	The suggested architecture generates clear routes for users to follow as they go to their destination, providing information along the way via audio and haptic feedback.	No, Only English	N/A
(121)	Lin et al., 2017	Simple Smartphone-Based Guiding System for Visually Impaired	intuitive and user-friendly, enhancing daily task convenience for visually impaired individuals. It significantly improves accessibility and usability, enabling efficient task performance with minimal effort	No, Only Chinese and English	A questionnaire was designed to see app’s effectiveness based 3 sectors overall usage, UI/UX and satisfaction. Out of 4, most users scored it above 3/5 in all 3 sectors mentioned above. Showing it as being effective and reliable.

**Table 4:** Definitions of user feedback—NMUD design, usability and universal usability for devices used by visually impaired.

Reference number	Authors and year	Target device for the visually impaired	Usability	Universal usability	User feedback—NMUD design
(77)	Kasthuri et al., 2017	Effective Guide System	A quick and efficient way to find out the most recent bus schedules, weather forecasts, and news.	Yes	N/A
(28)	Hela1 et al., 2001	Wireless Pedestrian Navigation System	Smart system which augments contextual information according to user preference, temporal constraints and dynamic obstacles, further improving usage.	No	N/A
(100)	Hoque et al., 2021	Blind Guard	Easy detection of objects nearby, easy to notify the user.	No	N/A
(78)	Mon et al., 2021	N/A	Easy to use and learn, useful in day-to-day life, satisfied users.	Yes	Fun to use, some need a technical assistant.
(32)	Huang et al., 2022	SAPSS (Smart Accessible Pedestrian Signal System) control box	Communicates with traffic light controller using analog signals, connects via Bluetooth to mobile phone and alerts the users.	No	When approaching a crossroads, anticipate automated notification, and when crossing a street, anticipate supplementary indications to keep the user on the correct path.
(29)	Harjumaa et al., 2011	Provides Medicine Management service	Enables users to recognize medicines, provide medicine information, convenient to use and learn.	Yes, the language of the content stored in NFC tag can be modified according to the country.	Easy to learn and use, limited functionality
(30)	Sung et al., 2013	Smart Cane	Detects and produces vibration alerts for obstacles in front of the user with a very extended obstacle detection range.	Yes	Better at detecting and avoiding obstacles than a smart cane, reported problems with the detection of floor-level obstacles
(79)	Huppert et al., 2021	GuideCopter	Facilitates with hand-object localization, precise haptic feedback and audio-based guidance.	Yes, but audio cues have to be modified according to language(region).	Systems are difficult to perceive and interpret and follow cues.
(108)	Khare et al., 2020	Voice to writing on paper using digital scribe.	Easy to write letters or fill forms with voice only.	Only English	No user testing done.
(109)	Devi et al., 2020	Head Wearable navigation and object avoidance system.	Further optimizations can make it a very useful headgear.	N/A	Very cluttered system in early stages and needs high optimization from a design perspective.
(110)	Ehrlich et al., 2017	Head-mounted Display (HMD)	Enhance functionality by magnifying visuals, enhancing contrast, and improving on peripheral detection to clarify environments, alongside audio feedback that substitutes visual cues with auditory information, aiding in navigation and daily tasks.	N/A	N/A

#### 3.2.4 Usability

In order to ensure high usability, the utilization of standard practices and notations, uniformity throughout the application, and making the interface as well as the actions by the users similar to the real world so that these actions come to them in a very natural manner are some of the suggested approaches. According to Table 3, which reviews over (*n* = 40) articles on apps for the CVI and PVI individuals, the prevailing opinion among users regarding the usability of the program is that it is perceived as user-friendly and effective in enhancing the convenience of their daily duties. It may be inferred that a mere (*n* = 11) applications possess universal usability, whereas the remaining applications lack this attribute primarily owing to limitations related to language. As discussed in Table 4, where we make an assessment on devices (*n* = 10) for the CVI and PVI, the majority of user comments on the usability have been that the application was found easy to use and successful in making their day-to-day tasks more convenient. It can be deduced that only five of the apps offer universal usability, and the rest do not, largely due to language constraints.

#### 3.2.5 Inclusive design/universal design/universal usability

The term “universal design” was devised at North Carolina State University in the context of architecture by Ronald L. Mace in the year 1985 (80). In order to implement universal design in a device or an application, both the macro view of the application as well as the micro view has to be taken into account (81). At the North Carolina State University’s Center of Universal Design, the experts including architects, product designers, engineers and researchers came up with seven principles of Universal design, namely, equitable use, flexibility in use, simple and intuitive, perceptible information, tolerance for error, low physical effort and size and space for approach and use (82–86).

#### 3.2.6 Standards and regulations for devices

A large number of standards exist for apps and devices, serving as rules that guarantee these items comply with specified requirements and are compatible with other products, for example, assistive applications and devices. The ISO 9999: 2022 standard sets forth a system for categorizing and labeling assistive products intended for individuals with disabilities (86). Regulations, on the other hand, refer to the legal requirements that vary according to different apps and devices and also by country. These administer the development, distribution, and the use of apps and devices in the user environment (87).

#### 3.2.7 Methodologies of evaluation—NMUD analysis of devices for visually impaired

Table 5 presents the methodology of NMUD analysis of devices for the visually impaired; it gives a comprehensive overview of scholarly publications (*n* = 10) that examine devices for CVI and PVI individuals. The primary objective of these articles is to evaluate essential features, including analysis methodologies for user interface (UI) and user experience (UX), advantages and disadvantages of the devices, and the specific devices targeted in the research. For NMUD analysis, testing tools like Selendroid (77), qualitative field trials (29), pre and post-trial interviews (29, 30), user evaluation testing (30), and ANOVA analysis (30) are all commonly used methods. According to users, the main benefits of any device are its affordability, customizability, scalability, compatibility with current solutions for the visually impaired (CVI and PVI), and the fact that wearable or easily transportable devices are small and portable. The key negatives cited in relation to any given application encompass significant financial expenses, challenges in comprehending its usage, the absence of power backup, and limited applicability across various scenarios. It can be observed that using voice recognition, location and haptics are the most common methods in which devices employ natural user interface in their overall architecture.

**Table 5:** Methodology of NMUD analysis of devices for visually impaired.

Reference number	Year	NMUD analysis methods	Pros	Cons	Target device	Use of NUI
(77)	2017	Selendroid Testing tool	Cost efficient and scalable to integrate Calendar, E-mail and services booking facilities.	No power backup; voice detection might be corrupted because of background noise; calendar, e-mail and booking of services not available; device might not work in case of network absence.	Effective Guide System	Using voice recognition.
(28)	2001	Street-crossing activities	The system is a supplement to other navigational aids like canes, blind guide dogs, and wheelchairs. It computes routes using user preferences, temporal constraints, dynamic obstacles, environmental conditions, and landmarks as well. It also gives the user the ability to add intelligence—as perceived by the CVI user—to the central server housing the spatial database.	Signal loss near tall buildings and tree canopies; the whole system might be heavy to wear everyday; not cost efficient; enough user testing not done.	Wireless Pedestrian Navigation System	Using real time navigation and sound.
(100)	2021	Software technology testing	Convenient, fast, reliable, and cost-effective, not bulky.	Authentication cannot be done using audio; stick cannot detect objects that are incoming from far to near and also objects that are not under a certain proximity.	Blind Guard	Using physical stick to detect and identify objects nearby.
(78)	2021	N/A	Device was bought and not invented.	Device was bought and not invented.	N/A	Uses Touch, Sound and Smell.
(32)	2022	Street-crossing activities	Minimalist design, can get exact location and seconds left to cross the street.	Will not work without smartphone, costly because it is a whole system	SAPSS (Smart Accessible Pedestrian Signal System)	Uses Touch and Sound
(29)	2011	Qualitative field Trial and interview post trial	Judge the practical impact on users in real-life situations, figure out the scope for improvement and evaluate market value.	Participants who do not meet the inclusion criteria took trial, 4–6 weeks of trial time not enough to perfectly gage user’s behavior.	Medicine Management Service	Uses NFC tag reader with speech output.
(30)	2013	Performance based prototype experience and evaluation, interviews (pre and post), ANOVA analysis	Qualitative and quantitative evaluation, deep insights into user’s experience	One-on-one depth interviews a time taking process, prototype development is costly and time consuming	Smart Cane	Uses tactile, haptics and audio signals.
(79)	2021	Comparative user study	Allows for a direct comparison between real-world guidance applications and collection of both subjective and objective data, scope for iterative feedback.	Small sample size with only 10 participants, limited scope of tasks and duration of use.	Drone-based haptic guidance interface	Uses Haptic Feedbacks, and audio cues.
(108)	2020	No testing done with CVI or PVI people.	Help directly convert voice to written text with readable words. More optimization can make it useful in many educational institutions.	The handwriting can be improved as it is very block style and hard to read. The speed and accuracy needs to be improved with more training. MIT app builder provides a very basic and non-engaging UI for the user.	Voice to written text application and hardware system.	Uses voice recognition.
(109)	2020	N/A	Wearable system of sensors and devices which help the user to navigate and monitor body basics like heartbeat, oxygen level.	Still at very early stages of development, a highly cluttered system of sensors which can easily confuse the user.	Multiple sensors working in sync to provide a navigation and basic health monitoring system.	Uses multiple sensors to get health statics.
(110)	2017	N/A	Enhanced accessibility by providing magnification, high contrast visuals, and auditory feedback which gives increased independence. Enhancement of central vision or night vision through image processing. They provide real-time, contextual information about the surrounding environment, aiding in navigation and object recognition. Also, they can be customized.	Expensive, technology can be too complex for users, HMD can be bulky which can cause physical discomfort, battery life dependency, can also cause unwanted attention, cannot see all colors.	Head-Mounted Display (HMD)	Voice input, trackpad input, eye/head sensing, gaze tracking, and hand sensing

#### 3.2.8 Methodologies of evaluation—NMUD analysis of apps for visually impaired

Table 6 presents the methodology of NMUD analysis of apps for the visually impaired. It reviews research articles (*n* = 44) on applications for the CVI and PVI, focusing on crucial aspects such as NMUD analysis methods, pros and cons, target devices and Use of NUI. Some of the widely employed approaches for NMUD analysis are questionnaires (78, 88, 89), usability studies (37), formative interviews (90–92), direct interviews (31, 91, 93–95), workshop interviews (14), and user evaluation testing (20, 96–98) and other methods (32, 49, 99–107). The major comments regarding the pros of any app, according to the users, are low cost, personalization, adaptability, availability in the language of their choice, and the app being platform-independent. The identified primary drawbacks associated with any application were its reliance on certain platforms, lack of personalization, significant latency, high cost, and the absence of user input in the final testing. It can be observed that touch, tactile, haptics and location are the most common natural user interfaces that were used in most of applications. These natural user interfaces help the CVI and PVI population interact more easily with the smartphone applications.

**Table 6:** Methodology of NMUD analysis of apps for visually impaired.

Reference number	Year	NMUD analysis methods	Pros	Cons	Target devices	Use of NUI
(37)	2015	Interface usability studies, on-road navigation testing	Low fidelity prototype and on-road testing is cost efficient	Absence of personalisation	BlindNavi (Navigation Application)	Uses Auditory, olfactory, and tactile clues
(111)	2012	N/A	iOS Vision Library developed which can be reused in future, portal open to contribution from developers	Not platform-independent	Low Vision Mobile Portal	Uses haptics and low vision wheel to control app.
(112)	2015	N/A	Low-cost and high-performance assistive system with seven readers	Not platform-independent	Voice Helper (Personal Digital Assistant)	TTS used for speech output.
(99)	2021	Remote study sessions	Available in English and Chinese, faster emoji availability	Not platform-independent, not supported real-time recognition owing to network difficulties, remote study sessions, uncontrolled network latency.	Voicemoji (Keyboard-based emoji entry)	Uses Speech to Text interface
(93)	2015	Pre-design and post-design interview	Cost effective, available in English and Bangla	Not platform-independent	mBraille (Braille Learning Application)	Uses haptics and gestures
(113)	2016	Formative Interviews	Capable of voice instructions and multi-touch interaction.	Not platform-independent	All Appointments (Appointment manager)	Uses Speech to Text interface
(94)	2018	Direct Interview	Easy to adapt, senses device shakes and performs tasks accordingly, reads out messages	Not platform-independent, controlled testing does not allow for observing users in a real life environment.	Intelligent Software Assistant	Uses Speech to Text interface
(39)	2019	Workshop interview	It is envisaged that the device will be available on mobile platforms like Android and iOS, be independent of web connectivity, allow for intermittent GPS inactivation, be reasonably priced, and be user-friendly.	Controlled testing does not allow for observing users in a real life environment.	TransmiGuia (Public transportation services assistant)	Uses Speech to Text interface and Geolocation navigation.
(90)	2020	Lab quiet and noisy environment testing	Operates on standard smartphones, allowing a wide range of consumers from different social strata to profit from using the system.	Not platform-independent; three commands’ recognition accuracy varies in both noisy and quiet environments; command misinterpretation; command response time for scanning text is 4 s; overall average latency is 1.875 s. The application is better at understanding user commands in a calm setting; nonetheless, face and object recognition are not supported.	DAVID	Uses Speech to Text interface
(96)	2013	User evaluation testing	Innovative idea, simplicity, and reasonably priced availability.	Not platform-independent, controlled testing does not allow for observing users in a real life environment.	Mobile Phone Dialler Application	Touch based input, screen divide in 6 areas.
(100)	2021	Software technology testing	Convenient, fast, reliable, and cost-effective.	Not platform-independent	Blind Guard	Ultrasonic camera, light and water sensors based blind stick. Works in coordination with application.
(78)	2021	7-point Likert scale questionnaire	Beneficial, simple to use, and content with the application.	High cost, a shortage of samples due to COVID, some of them requiring technical support to use the application—appropriate techniques and technologies should be adopted.	3DOHA application	Touch, Sound and smell used.
(32)	2022	Street-crossing activities	Platform independent, minimalist design	Only available in Chinese language	SAPSS (Smart Accessible Pedestrian Signal System)	Touch, Sound and Signals
(101)	2015	Remote multi session evaluation using a simulated work task.	Cost efficient; Allows to observe the users in a very real-time environment; Users give a very unbiased review; allows to examine an assorted group of users.	Participants were not familiar with the interface; prior training required; not enough time	Web search interface for visually impaired	Using Speech to text.
(102)	2016	Lead-in usability evaluation (based on task performance) and *ex post facto* interview with users.	Qualitative (user reactions and interview) and quantitative (calculate different measures of user performance).	Prototype development is costly and time taking.	Application for accessing museum collections.	Uses audio, tactile and haptics.
(103)	2015	Indoor and outdoor user testing, pilot studies with people who are CVI or PVI, and a final assessment by industry professionals.	Found user behavior in different contexts, expert evaluation is quick	No user involvement in final evaluation	(Android application-uses a smartphone connected to a smartwatch)	Audio and Haptics used.
(88)	2011	Questionnaire (to gather user level and feedback of prototype) and working with prototype	Pre and post questionnaires to work with prototypes enable to properly define a goal and evaluate results.	Controlled testing does not allow for observing users in a real life environment.	E-learning device	Combines Braille and TTS.
(91)	2018	Application test by users, interview	Implemented as a part of the Double -diamond approach, ensures that you identify and understand the foundational problem correctly.	Difficult to find the best set of candidates for the test and trial, working prototype required, time-taking.	Braille learning application	Dynamic virtual braille keyboard and Voice input
(49)	2019	Pretest,post-test (UX tests),task observations and video and audio recording.	Ensures better feedback and satisfactory ux the for users	Time consuming process	Android application	Uses Speech to text and Text to Speech
(92)	2019	Formative interviews, user-elicitation studies, user studies for evaluation of the prototype	Distinctly recognize user expectations and gather adequate user feedback	Time consuming, development of prototype is costly	Interaction technique implemented in smartphones	Uses touch gestures.
(95)	2018	User-survey via interviews through both in person and phone.	Clarity on user requirements and expectations prior to development.	No adequate feedback on the product developed is gathered.	Android Application	Uses speech to run various commands.
(89)	2017	Questionnaire (pretest), interviews (post-test)	Expeditious answers, gain insights in both subjective and objective manner from varied groups of people.	Interviews require a lot of pre-work, cannot be objective and time consuming	Navigation assistant	Uses audio and vibrational haptics.
(105)	2021	Literature review	Quick, cheap	No user involvement	Online shopping mobile application	Uses audio and spatial navigation.
(59)	2021	N/A	N/A	N/A	Navigation Support Application.	Audio and navigation
(114)	2019	N/A	N/A	N/A	Object Recognition Application.	Uses image recognition and audio
(100)	2021	Requirements gathering from CVI, guard and the administrator, organized reviews post generation of test reports.	Developed system takes care of the the user needs and concerns and appropriate feedback	Time consuming, building a prototype for testing is costly.	Communication and navigation support application	Uses audio including alert sounds and speech; location
(97)	2019	Analysis of user testing (performing tasks) of the prototype	Gain sufficient insights on user feedback and suggestions	Time consuming	Navigation Support Application	Audio.
(20)	2018	Testing product live with 3 users.	Provides a full solution for home automation for visually impaired. Lights and appliances are in full control.	Lack clarity of various services in UI. lack of Interface partitioning in logical sections.	Home Automation software	Various sensors and buttons controlled by user.
(115)	2021	N/A	Easy to use and clear audio output describing the image taken. AI model used for captioning.	To get appropriate images we will need a lot of images and heavy CPU computation. Flutter TTS is not heavily accurate for large scale usage.	Image Caption provider for visually impaired with smartphone.	Uses image recognition
(106)	2021	Multiple rounds of image/object recognition testing were done to verify working of the product.	Currency detection and identification, Information extraction and currency denomination, Obstacle Differentiation and Identification, Distance Measurement, Face Recognition	Recognition of faces and objects in low light requires a good quality camera, which may/may not be present on the users phone. Everything depends on the user’s phone configuration.	Day to Day image/object recognition.	Uses sensors and audio feedbacks.
(31)	2017	Tested live at supermarket and 5 users with 95% success rate	The visually impaired do not need to learn a new set of navigational skills in order to utilize the system. A cognitive mind map provides the number of steps needed to reach each shelf and obtain the desired objects.	Sometimes barcodes do not work properly. Frequent changes in product locations might cause issues, app is useless if the server is down, app is available only in English.	Shopping Navigation.	Location, navigation, audio and haptics.
(116)	2020	N/A	Easily help users to fill form fields with clearly visible buttons for each form field having Text To Speech functionality.	Not all forms are easy to represent in a voice based format, most require a lot of information from the user in different formats. How will the user ensure all the details printed are right. Ability to re-speak/edit the form details. No support for different languages. Very basic and bad UI used.	Form Filling via voice input.	Physical buttons and speech to text.
(117)	2010	N/A	Aimed at resolving the problems with usability and accessibility in electronic publication. The procedure can be simplified and time saved by doing a number of actions semi-automatically.	Currently, we can only offer help for straightforward textbooks; however, efforts are being made to secure support for books in the sciences like physics, chemistry, and maths.	Converting physical books to E-Books for easy reading and listening via text to speech.	None, only used for converting book to E Book format.
(118)	2020	N/A	Multi Language support for messaging and emails.	Material UI rules not used in the application Through user testing not done. Mobile databases are not recommended to be used in the long term.	User messaging and email for CVI and PVI individuals with voice commands.	TTS, Speech to Text and location navigation.
(98)	2011	Analysis of user testing	After the learning curve users feel very comfortable and fluent with the touch areas and patterns.	Cannot implement special characters in this. Needs a good amount of learning curve for users to get used to the application.	Special application for helping users type with touch patterns in phone screen.	Various touch gestures are used.
(62)	2012	N/A	Helps users to get information about different bus timings so that they can efficiently reach the bus stop on time and be able to independently travel.	Not synced in real time with buses to get updates about bus delays. ABLE Transit a Web application is the fact that all browsers and devices offer a variety of HTML5 APIs, which might result in inconsistent user experiences.	Application to help users independently travel via bus by providing bus timing information.	Audio and GPS Location tracking.
(107)	2019	A preliminary test session was performed to check the accessibility and the provided functions.	Provides a wide list of services for day to day activities. All services are decided based on questionnaires done with visually impaired people for accurate services.	The only issue was that the app’s sharing services feature was difficult to discover and could not be voice controlled.	A general purpose application with various services decided based on a user questionnaire.	Audio Navigation.
(119)	2015	N/A	Accurately identifies a number of items and also helps in color recognition using the phone’s camera.	It’s hard for a visually impaired user to train the system, Material UI is not used, UI does not cope well with new screen sizes and designs.	Object & Color recognition system.	Image recognition used.
(122)	2012	Analysis of real time user testing on visually impaired people	Smartly understand text written and convert it to speech output. Lines of text that cross the boundaries of any particular picture frame can be read without any problems.	System has to be significantly upgraded before it is useful. The user had to painstakingly scan wide regions in order to find text in an unidentified spot. The experiment was done in low-light settings with sluggish processing rates, motion blur, and high texture areas, which made it necessary for the user to scan slowly.	OCR (Optical Character Recognition) system.	Image and Text recognition interface.
(65)	2014	N/A	Take input from crowdsourcing and merge it for visually impaired users to avoid getting hurt on stairs and potholes.	System not yet fully designed for completely visually impaired users and needs several GUI clicks to work. Still in a very early stage.	Navigation with staircase and pothole warnings.	Audio and Haptics used along with navigation
(120)	2021	20 individuals volunteered to test our navigation system. Each user is given the following three tasks: using a remote, operating a treadmill, and clicking a button on a webpage. One on one interviews were also conducted.	(16 out of 20) preferred the system because it provides a very easy way to navigate finger placement on various devices like TV remote, Treadmill, etc. Guides users using clear voice communication.	The implementation of object detection was one restriction. Currently, mobile phones cannot use more sophisticated, precise machine learning techniques like Faster-RCNN. This can occasionally cause a response delay, which degrades system performance. In case of many things in the environment, too much voice info confuses the user.	Voice guiding application to help identify button positions on various devices.	Gestures and Voice commands used.
(67)	2020	N/A	Provides multiple services like Object Recognition, News Reader, Navigation, Alarm and Reminder with voice based commands	No support for iOS Can recognize only 80 objects. Better screen space utilization. A lot of screen space is wasted currently	Voice guiding application to help in Object Recognition, News Reader, Navigation, Alarm and Reminder.	Uses voice/audio and location.
(68)	2021	6 individuals alternately navigated the field while wearing blindfolds and watched the navigations on the monitoring tool.	Very minimal UI, near accurate location of potholes to help users not get hurt, getting to the current location quickly.	In confusing roads, the system voice commands can be really confusing and lead to an accident. Not so easy to understand at first and learn efficiently.	Android Application helping users in multiple day to day activities.	Uses voice/audio and location to avoid potholes.
(121)	2017	A questionnaire was used on live subjects using the application to understand app’s review overall usage, UI/UX, notify frequency satisfaction.	Realtime image recognition and easy to use UI and UX. Also gives approximate distance to an object. Works like an effective navigation agent.	Only available in Android and written in now less preferred JAVA instead of Kotlin. Only in 2 languages. Has a limited image recognition ability in OFFLINE mode. Does not adhere to Material UI standards for UI and UX.	Android Applications with connectivity to 4G internet for optimum usage.	Audio, Haptic feedback, and User Location

## 4 Open challenges

One of the significant challenges faced in designing devices for visually impaired individuals is integrating new technologies with existing practices, addressing battery life limitations and slow real-time processing resulting in slower reaction times. Safety concerns are also a crucial factor that designers must consider when developing these devices. However, the functionality of these devices is limited, and there is ample scope for improvement. Additionally, the user interface and user experience design of these devices present their own set of challenges, including high cognitive overload, extensive learning requirements, non-catchy interfaces, and limited functionality. Addressing these challenges is essential for ensuring that visually impaired individuals can use these devices efficiently and safely, ultimately leading to a higher quality of life. Furthermore, there are several open challenges in designing apps and devices for visually impaired individuals, including compatibility with assistive technologies, customization options, feedback mechanisms, and user support availability. Overcoming these challenges is crucial for providing visually impaired individuals with greater independence and a higher quality of life, and continued research and development in this area are necessary.

### 4.1 Open challenges—NMUD design of devices for visually impaired

**Figure 4:**
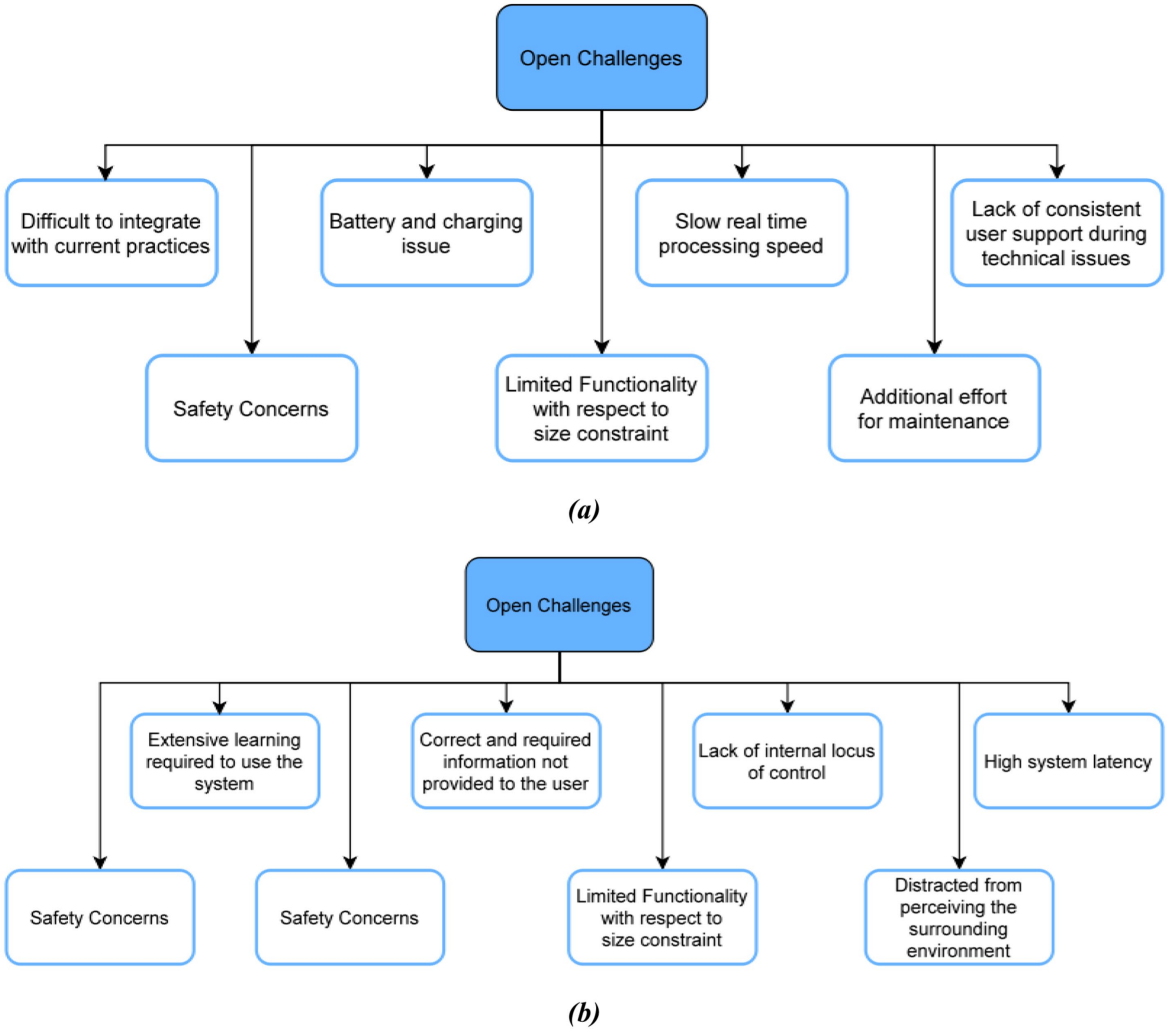
**(a)** Open challenges—NMUD of devices for visually impaired; **(b)** open challenges—NMUD of apps for visually impaired.

Figure 4a illustrates the open challenges concerned with the NMUD of devices for the visually impaired, such as frequent charging and battery issues, it is hard to achieve high processing speed which can provide output in very little time after processing all the input from the environment. There are a lot of safety concerns with these devices as the user is performing all his/her major tasks based on the inputs provided by the device, and any error in output, navigation, or response can cause accidents. There is also limited functionality that is possible with respect to size constraint. Additionally, we need to provide constant customer and hands-on support for the device. Figure 4b portrays the open challenges related to the NMUD of Apps for the Visually Impaired.

### 4.2 Open challenges—NMUD of apps for visually impaired

The open challenges associated with the NMUD of apps for the visually impaired are shown in Figure 4b. The device may not provide accurate results every time due to several distractions in the environment and technical glitches. The user needs to go through a long learning curve for most applications to learn all the gestures or voice commands to operate the application. Many times, there is also a lack of internal locus of control.

## 5 Discussion

### 5.1 Synthesis, interpretations and comparisons of results

This comprehensive review sheds light on several critical findings concerning the commercialization and usability of devices and applications designed for individuals with Complete Visual Impairment (CVI) and Partial Visual Impairment (PVI). As indicated in Table 1, there is a wide array of innovative devices developed for visually impaired users. Despite their innovation, a significant gap in commercialization persists. Many devices are geared toward healthcare, navigation, and communication, yet they often overlook essential areas such as social inclusion, employment opportunities, financial inclusion, and independent living. This gap underscores the necessity for more comprehensive solutions that address the diverse needs of visually impaired individuals.

Moving to applications, Table 2 elaborates on the usability and functionality of various apps designed for the visually impaired. A striking observation is that out of 43 reviewed applications, 24 have not yet been commercialized, which suggests a gap between development and market availability. The apps are primarily designed to provide auditory, tactile, or Braille feedback, enhancing navigation, communication, and daily task management for visually impaired users. This underscores the potential of such applications to significantly improve the quality of life for these individuals by making smartphones more accessible and versatile in performing a wide array of tasks.

Tables 3, 4, which examine apps and devices respectively, reveal that a notable proportion of articles (26 out of 53) lack thorough discussions on user feedback. This is a significant oversight because user feedback is crucial for evaluating the effectiveness of these solutions. Additionally, the usability of many proposed applications and devices is limited by language constraints, restricting their universal applicability. This highlights the importance of incorporating language inclusivity in the design and development of assistive technologies.

Tables 5, 6 present a variety of Natural User Interfaces (NUI) that are essential to the advancement of assistive technologies because they provide users with simple and convenient interactions. Gesture recognition is one of them, enabling users to interact with devices using their own natural movements. Because it can be tailored to identify motions that are appropriate for the user’s skills, this type of interaction is very helpful for people who have physical limitations. While gesture recognition offers a high level of user involvement, its accuracy and responsiveness might vary depending on the technology and environment. As such, gesture recognition requires rigorous calibration and user training to attain maximum effectiveness.

Another common NUI type included in these Tables 5, 6 is voice command interfaces. It is especially helpful for visually impaired people since they allow spoken language control over equipment and information access. Voice commands are an intuitive interface that requires little physical involvement, making it usable by a variety of users. However, elements like background noise, language obstacles, and the quality of speech recognition can affect how successful voice command systems are. Voice commands are emphasized as an essential NUI element that improves usability by offering a hands-free and eyes-free interaction option, despite these difficulties.

Because they are recognizable and simple to use, touch-based interactions which are typically seen in smartphones and tablets are also widely employed in assistive devices. These interfaces provide a clear, generally accessible design and let users interact with gadgets with easy taps, swipes, and pinches. According to the papers cited in Tables 5, 6, touch-based NUIs are preferred due to their ease of use and the abundance of touch-enabled devices on the market. But obstacles including sensitivity problems, screen accessibility, and the requirement for visual input might reduce their usefulness, especially for people with certain impairments.

Similarly, Table 6 shows that 32 out of 44 articles provide insights into design and user experience analysis. The articles employ various NMUD analysis methods, including questionnaires, usability studies, formative interviews, direct interviews, and user evaluation testing. The primary advantages highlighted include low cost, personalization, adaptability, and availability in multiple languages. However, drawbacks such as reliance on specific platforms, lack of personalization, significant latency, high costs, and absence of user input in final testing are noted. These detailed discussions emphasize the necessity of thorough NMUD analysis in creating effective assistive applications.

When compared to other review papers, this study distinguishes itself by offering a detailed analysis of various applications and devices for the visually impaired from a NMUD perspective. It addresses multiple open problems and future challenges, relating them to each application and device. Other review papers often lack comprehensive statistics, such as those shown in Figures 2b, 3a,b, which illustrate the PRISMA flow diagram for article selection, the distribution of articles by year, and the geographic distribution of research, respectively. The analysis of Tables 1–6 provides a nuanced understanding of the current state of NMUD for applications and devices aimed at visually impaired users. The study meticulously reviews the functionality, usability, and user feedback associated with these technologies, offering a detailed and critical assessment that is often missing in other review papers.

### 5.2 Current review limitations

We have identified some key limitations of the review study such as (i) NMUD preferences and trends are ever evolving and change every couple of years, so sometimes it is not feasible to do an end-to-end comparison between articles of different years of publication. (ii) Language Barrier played a major role in the filtering of articles, since we have limited the scope of only English articles, it is very well possible that this article missed worthy articles just because they were not in English. (iii) The Generalization Challenge was present throughout the review as the needs and preferences of the visually impaired population differ with different age groups, sex, and other demographic criteria, this combined with different levels of impairment increases the complexity. (iv) All the reviews were conducted fully on existing work of other authors, so it assumes all of their assumptions and biases as a requirement for the literary work. No professional or personal opinion was included to evaluate to moral or ethical value of the existing reviews (Table 7).

**Table 7:** Open challenges—NMUD of devices for visually impaired.

Reference	Challenge 1	Challenge 2	Challenge 3	Challenge 4	Challenge 5	Challenge 6	Challenge 7
(77)	**×**	**✓**	**×**	**×**	**✓**	**×**	**✓**
(28)	**×**	**✓**	**✓**	**✓**	**×**	**×**	**×**
(100)	**×**	**✓**	**×**	**×**	**×**	**×**	**✓**
(78)	**✓**	**✓**	**×**	**×**	**×**	**✓**	**×**
(32)	**✓**	**×**	**×**	**✓**	**✓**	**×**	**✓**
(29)	**✓**	**×**	**×**	**✓**	**×**	**✓**	**✓**
(30)	**×**	**✓**	**✓**	**✓**	**×**	**×**	**✓**
(79)	**✓**	**✓**	**✓**	**✓**	**×**	**✓**	**×**
(108)	**✓**	**✓**	**✓**	**×**	**✓**	**×**	**×**
(109)	**✓**	**✓**	**×**	**×**	**✓**	**×**	**✓**
(110)	**×**	**✓**	**×**	**✓**	**×**	**×**	**×**

✓: Available, ×: Not Available. **Challenge 1**: Difficult to integrate with the existing practices; **Challenge 2**: Battery issues; **Challenge** 3: Real-time processing takes longer time resulting in slower reaction time; **Challenge 4**: Safety concerns; **Challenge 5**: Limited functionality with a scope for improvement; **Challenge 6**: Require additional effort for maintenance; **Challenge 7**: Does not provide the required level of support to the user.

### 5.3 Future research directions

**Figure 5:**
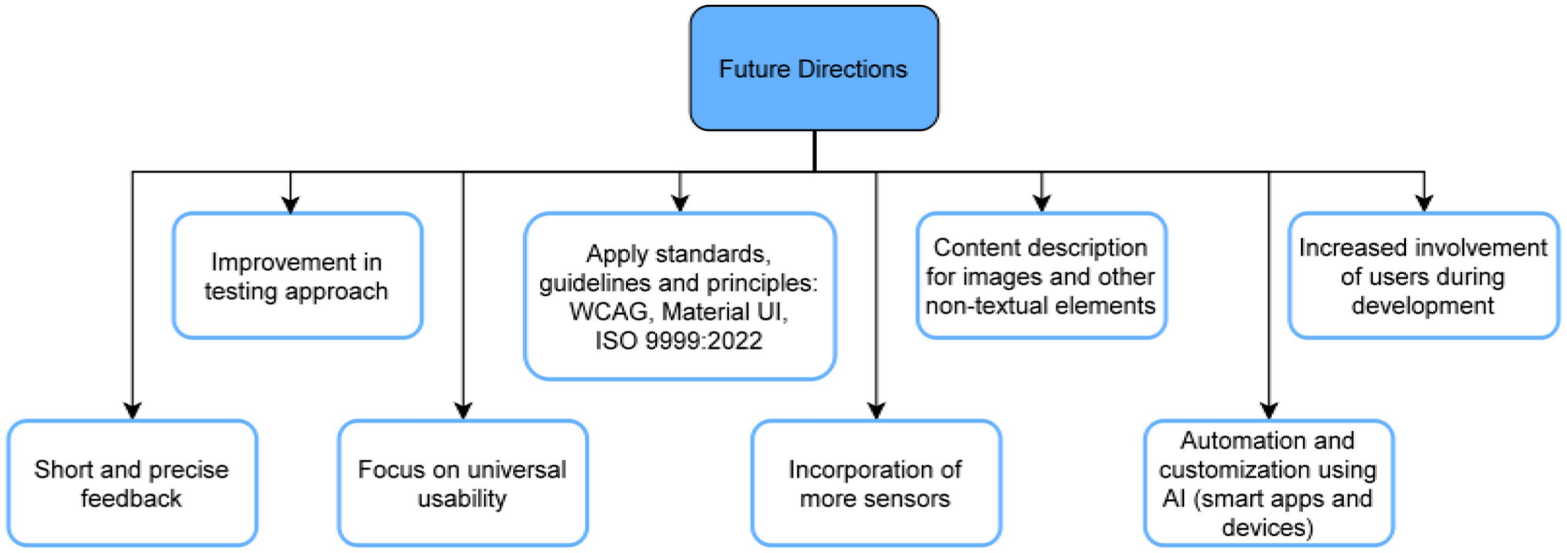
Future research directions.

From the detailed analysis of all the articles and comprehensive review of recent trends, there are many fields and directions in which exploration can be done by researchers as well as developers of apps and devices. Figure 5 represents the future research directions. Since this study is targeted toward visually impaired users, apart from voice feedback, non-speech sounds should be included more to improve receptivity and provide more information when interacting with screen-readers. The audio instructions must be minimal, short, and precise to ensure they do not cognitively overburden the user. Our review has shown that if CVI (Completely Visually Impaired - Blind people) and Partially Visually Impaired (CVI and PVI) users are included in all stages of development of the system and not just at the time of final user testing, they can provide very crucial input to the process ensuring high efficiency and usability. There must also be a facility for seamless integration with supporting Application Programming Interfaces (APIs) and other assistive technologies for web and mobile applications (Table 8).

**Table 8:** Open challenges—NMUD of apps for visually impaired.

Reference	Challenge 1	Challenge 2	Challenge 3	Challenge 4	Challenge 5	Challenge 6	Challenge 7	Challenge 8
(37)	**✓**	**×**	**✓**	**✓**	**×**	**✓**	**✓**	**✓**
(111)	**✓**	**✓**	**×**	**×**	**×**	**×**	**✓**	**✓**
(112)	**✓**	**✓**	**×**	**×**	**✓**	**✓**	**×**	**✓**
(99)	**×**	**×**	**×**	**✓**	**✓**	**×**	**×**	**✓**
(93)	**×**	**×**	**×**	**×**		**×**	**×**	**✓**
(113)	**✓**	**✓**	**✓**	**✓**	**✓**	**✓**	**✓**	**✓**
(94)	**×**	**×**	**×**	**×**	**×**	**×**	**×**	**✓**
(39)	**×**	**×**	**×**	**×**	**×**	**×**	**×**	**✓**
(90)	**×**	**×**	**✓**	**✓**	**✓**	**✓**	**×**	**✓**
(96)	**×**	**×**	**×**	**✓**	**×**	**×**	**✓**	**✓**
(100)	**×**	**×**	**×**	**×**	**×**	**×**	**×**	**✓**
(78)	**×**	**×**	**×**	**✓**	**✓**	**×**	**×**	**✓**
(32)	**×**	**✓**	**✓**	**✓**	**×**	**×**	**✓**	**✓**
(101)	**✓**	**✓**	**×**	**✓**	**✓**	**×**	**✓**	**×**
(102)	**✓**	**×**	**×**	**×**	**×**	**✓**	**×**	**✓**
(103)	**✓**	**✓**	**×**	**×**	**✓**	**✓**	**×**	**×**
(88)	**×**	**×**	**✓**	**×**	**×**	**×**	**✓**	**×**
(91)	**×**	**✓**	**✓**	**×**	**×**	**×**	**×**	**✓**
(49)	**×**	**×**	**✓**	**✓**	**✓**	**×**	**×**	**×**
(92)	**✓**	**×**	**×**	**×**	**✓**	**✓**	**✓**	**×**
(95)	**×**	**×**	**×**	**×**	**×**	**✓**	**✓**	**✓**
(89)	**×**	**✓**	**×**	**×**	**✓**	**×**	**×**	**✓**
(105)	**×**	**×**	**×**	**×**	**×**	**×**	**×**	**✓**
(59)	**×**	**×**	**✓**	**×**	**✓**	**×**	**×**	**×**
(114)	**×**	**×**	**✓**	**×**	**✓**	**×**	**×**	**✓**
(100)	**×**	**×**	**×**	**×**	**×**	**×**	**×**	**×**
(97)	**×**	**×**	**×**	**×**	**✓**	**×**	**×**	**✓**
(20)	**×**	**✓**	**✓**	**×**	**×**	**×**	**✓**	**×**
(115)	**×**	**×**	**✓**	**×**	**✓**	**×**	**✓**	**×**
(106)	**×**	**×**	**✓**	**×**	**×**	**×**	**×**	**×**
(31)	**×**	**×**	**✓**	**×**	**✓**	**×**	**✓**	**×**
(116)	**×**	**×**	**✓**	**✓**	**✓**	**×**	**✓**	**✓**
(117)	**×**	**✓**	**×**	**×**	**×**	**×**	**×**	**✓**
(118)	**×**	**×**	**✓**	**×**	**✓**	**×**	**✓**	**×**
(98)	**×**	**✓**	**✓**	**×**	**×**	**×**	**×**	**✓**
(62)	**×**	**×**	**✓**	**×**	**✓**	**×**	**✓**	**×**
(107)	**×**	**✓**	**×**	**×**	**×**	**×**	**×**	**✓**
(119)	**×**	**✓**	**✓**	**×**	**×**	**×**	**✓**	**×**
(122)	**×**	**✓**	**✓**	**×**	**✓**	**✓**	**×**	**×**
(65)	**×**	**×**	**×**	**×**	**×**	**×**	**×**	**×**
(120)	**✓**	**✓**	**✓**	**×**	**✓**	**×**	**✓**	**×**
(67)	**×**	**×**	**✓**	**×**	**✓**	**×**	**✓**	**✓**
(68)	**×**	**×**	**×**	**×**	**✓**	**✓**	**×**	**×**
(121)	**×**	**×**	**×**	**✓**	**✓**	**×**	**✓**	**✓**

✓: Available, ×: Not Available. **Challenge 1:** High cognitive overloads on the user; **Challenge 2:** Extensive learning required to learn to use the system; **Challenge 3:** Correct and required information not provided to the user; **Challenge 4:** Lack of internal locus of control; **Challenge 5:** High system latency; **Challenge 6:** Users are distracted from perceiving the real-time surrounding environment information; **Challenge 7:** Non-catchy and annoying interface with unsatisfactory design; **Challenge 8:** Limited functionality.

Web application developers must familiarize themselves with the Web Content Accessibility Guidelines (WCAG) that serve the specific purpose of making the web accessible to people with disabilities. The practical principles of e-accessibility must also be applied while developing websites, programs, etc. Material UI, a well-known library based on React, which follows Google’s Material Design principles, is highly beneficial for developers. It provides pre-built UI components that simplify the development process and result in a consistent visual style throughout the application, saving time and effort. Future efforts will focus on creating features and components that make editing tasks easier and facilitate web-based collaboration. A web-based application for document conversion, adaptation, collaboration, and sharing will be considered alongside Microsoft Word and OpenOffice.org Writer add-ons to help accomplish this goal.

There needs to be further work to utilize haptic feedback in a much wider range of situations and easy detection of complex and emergent devices, especially for smart cane devices. The development in the previously mentioned arena would also improve. GuideCopter, a tethered haptic drone interface, has the potential to enhance the user’s immersion in virtual and augmented reality. To give CVI and PVI users the greatest extent feasible an engaging experience similar to that of people who are not visually impaired users, colored text and other visual cues like bold, italic, flickering, or highlighted text can be translated into content description. EarTouch and similar devices can be given artificial intelligence so that they can automatically regulate the volume and switch from ear speakers to loudspeakers based on the user’s position in relation to the screen and the surrounding environment.

In future research studies, a wider range of participants with diverse factors that include chronological age, cultural background, touch-screen experience, and mobile device fluency should be considered, and their input should be incorporated into the development process. There should also be more focus on personalization and development of tools that allow for easy end-user customization to make the end products well-suited to the needs of each user. The architecture of these apps must be broadened to encompass multi-language functionality, including local and regional languages, to reach a larger audience.

It is crucial to acknowledge the significance of the social surroundings in the adoption of technology in future research. Potential future projects could concentrate on adding captions and audio descriptions to videos to provide ongoing, real-time information to individuals who are visually impaired (10). To enhance the navigation systems, incorporating a vast array of sensors can aid in object detection and health monitoring.

For the applications, there must be an attempt to make them standalone to a high degree to reduce the reliance on the user’s phone hardware. The applications should also be available on both Android and iOS. Creating something as simple as a help button will guide the users in case of an emergency or if they get lost within the application and help improve the internal locus of control. To have a high-performing and reliable database for mobile applications, it is advisable to utilize appropriate database technology like SQLite or Realm, enhance query optimization, reduce data transfers, introduce caching, keep track of performance, regularly maintain the database’s compactness, and, if necessary, use a remote database for extensive data or multiple user scenarios. The “view. setContentDescription,” which is an Android-specific function, must be used liberally to optimize the user experience, since without the content description set, the screen reader will just read the text.

It is recommended that the apps and devices undergo testing by a substantial group of visually impaired individuals to generate a larger dataset. A comprehensive examination should be conducted to investigate the testing scenario in depth. This will generate valuable information that can be utilized to enhance the testing process in a more analytical manner. Extension of the testing period will enable a deeper insight into the user population and their medication usage habits (HearMe).

### 5.4 Key takeaways

In this work, we first emphasize how important it is for users who are visually impaired to have access to audible and tactile feedback in the form of elevated buttons, vibrations, and aural signals. In our second section, we address the enduring issues of pricing and accessibility, pointing out that high costs, restricted functionality, and system latency are the main obstacles to universal access. Moreover, we support a user-centered design methodology that puts the unique requirements and preferences of visually impaired users first and guarantees a smooth interaction with assistive technology and online content accessibility rules. Furthermore, the significance of integrating with pre-existing technologies is underscored, as it capitalizes on recognizable interfaces to mitigate the learning curve and augment acceptance among this population. Together, these observations show the complex opportunities and challenges involved in creating NMUD that is inclusive and empowering for people with visual impairments.

## 6 Conclusion

In this review article, the taxonomy of the key design elements that have been considered in the design of apps and devices for visually impaired individuals is presented. Various review articles have been compared to analyze the depth of exploration that each article has undertaken in this domain. Everyday life usage, transmission of information, and the working of each application and device have been discussed in our collected set of articles. A brief review of various terminologies like HCI, UI, UI Design, UX & UX Design, Usability, and Universal Usability has been provided. Standard guidelines for making applications and devices for visually impaired users were also discussed. Apart from usability, universal usability, and user feedback, the benefits and detriments of proposed/implemented ideas were examined for respective articles. Developers and designers can utilize the discussed open problems by using our future directions. Frequently observed open problems were battery issues, lack of internal locus of control, high system latency, and limited functionalities. Developers and designers willing to develop novel approaches to cater to the needs of CVI and PVI users can utilize this review as a roadmap/guide as it lays out precisely the working, usability, and user feedback of a vast array of apps and devices and come up with solutions that can be based on the listed methods. Researchers in the field of NMUD will find this as a comprehensive guide to the areas in which they need to work in the future to address the mentioned open challenges. Despite the extensive scope of the NMUD field, there are several unresolved issues, indicating ample room for advancement in this area.

## Appendix A

**Table A1:** List of abbreviations used in the manuscripts along with their full form.

Acronym	Definition
UI	User Interface
UX	User Experience
NUI	Natural User Interface
NMUD	NUI, Multi-sensory and UX Design
WCAG	Web Content Accessibility Guidelines
E-accessibility	Electronic Accessibility
CUI	Character User Interface
GUI	Graphical User Interface
HCI	Human-Computer Interaction
ISO	International Organization For Standardization
VR	Virtual Reality
AR	Augmented Reality
CVI	Completely Visually Impaired
PVI	Partially Visually Impaired
WHO	World Health Organization
PRISMA	Preferred Reporting Items for Systematic Reviews and Meta-Analyses
N	Number
O&M	Orientation and mobility
GPS	Global Positioning System
NFC	Near-Field Communication
SRE	Speech Recognition Engine
GIS	Geographic Information System
3D	Three-dimensional
SAPSS	Smart Accessible Pedestrian Signal System
STT	Speech-to-Text
LVA	Low vision aid
OCR	Optical Character Recognition
API	Application Progamming Interface
NBD	Nodes-based dialler
NLP	Natural Language Processing
SSD	Single Shot Detector
IOT	Internet of Things
SMS	Short Message Service
GTFS	General Transit Format Specification
TTS	Text-To-Speech
HMD	Head Mounted Display
